# Preparation and PET/CT imaging of implant directed ^68^Ga-labeled magnetic nanoporous silica nanoparticles

**DOI:** 10.1186/s12951-023-02041-8

**Published:** 2023-08-17

**Authors:** Andras Polyak, Heidi Harting, Nina Angrisani, Timo Herrmann, Nina Ehlert, Jessica Meißner, Michael Willmann, Silav Al-Bazaz, Tobias L. Ross, Jens P. Bankstahl, Janin Reifenrath

**Affiliations:** 1 NIFE – Lower Saxony Centre for Biomedical Engineering, Implant Research and Development, Stadtfelddamm 34, 30625 Hannover, Germany; 2 Department of Prosthetic Dentistry and Biomedical Materials Science, Hannover Medical School, Carl-Neuberg-Straße 1, 30625 Hannover, Germany; 3 Department of Nuclear Medicine, Hannover Medical School, Carl-Neuberg-Straße 1, 30625 Hannover, Germany; 4 Clinic for Orthopedic Surgery, Hannover Medical School, Carl-Neuberg-Straße 1, 30625 Hannover, Germany; 5 Institute for Inorganic Chemistry, Leibniz University Hannover, Callinstraße 9, 30167 Hannover, Germany; 6 Department of Pharmacology, Toxicology and Pharmacy, University of Veterinary Medicine Hanover, Foundation, Buenteweg 17, 30559 Hannover, Germany

**Keywords:** Drug delivery systems, Magnetic targeting, Macrophage depletion, PET imaging, PEGylation, Core–shell nanoparticles, Implant imaging

## Abstract

**Background:**

Implant infections caused by biofilm forming bacteria are a major threat in orthopedic surgery. Delivering antibiotics directly to an implant affected by a bacterial biofilm via superparamagnetic nanoporous silica nanoparticles could present a promising approach. Nevertheless, short blood circulation half-life because of rapid interactions of nanoparticles with the host’s immune system hinder them from being clinically used. The aim of this study was to determine the temporal in vivo resolution of magnetic nanoporous silica nanoparticle (MNPSNP) distribution and the effect of PEGylation and clodronate application using PET/CT imaging and gamma counting in an implant mouse model.

**Methods:**

PEGylated and non-PEGylated MNPSNPs were radiolabeled with gallium-68 (^68^Ga), implementing the chelator tris(hydroxypyridinone). 36 mice were included in the study, 24 mice received a magnetic implant subcutaneously on the left and a titanium implant on the right hind leg. MNPSNP pharmacokinetics and implant accumulation was analyzed in dependence on PEGylation and additional clodronate application. Subsequently gamma counting was performed for further final analysis.

**Results:**

The pharmacokinetics and biodistribution of all radiolabeled nanoparticles could clearly be visualized and followed by dynamic PET/CT imaging. Both variants of ^68^Ga-labeled MNPSNP accumulated mainly in liver and spleen. PEGylation of the nanoparticles already resulted in lower liver uptakes. Combination with macrophage depletion led to a highly significant effect whereas macrophage depletion alone could not reveal significant differences. Although MNPSNP accumulation around implants was low in comparison to the inner organs in PET/CT imaging, gamma counting displayed a significantly higher %I.D./g for the tissue surrounding the magnetic implants compared to the titanium control. Additional PEGylation and/or macrophage depletion revealed no significant differences regarding nanoparticle accumulation at the implantation site.

**Conclusion:**

Tracking of ^68^Ga-labeled nanoparticles in a mouse model in the first critical hours post-injection by PET/CT imaging provided a better understanding of MNPSNP distribution, elimination and accumulation. Although PEGylation increases circulation time, nanoparticle accumulation at the implantation site was still insufficient for infection treatment and additional efforts are needed to increase local accumulation.

**Supplementary Information:**

The online version contains supplementary material available at 10.1186/s12951-023-02041-8.

## Background

Implant infections especially in the form of periprosthetic joint infections (PJI) are a major threat in orthopedic surgery [1, 2]. PJI treatment is resource- and cost-intensive [3] due to long-term antibiotic treatment and often-required additional surgery [4]. Over the last years, several studies showed that the incidence of PJI is increasing [1, 4, 5], especially because the total number of arthroplasties is growing [6, 7]. Bacteria causing PJI can reach implants on different ways. Inoculation of microorganisms during surgery is still a major cause and the virulence of the microorganisms determinates at what time point clinical signs become evident [7]. Apart from that, there is a life-long risk for colonization of implants with haematogenous spread bacteria after a primary infection somewhere else in the body [7]. The major problem regarding antibiotic treatment of PJI is that bacteria adhere to the implant surface and tend to form biofilms. Biofilm makes them a lot more resistant to antibiotic treatment than their planktonic form [7, 8], leading to the problem that necessary local antibiotic concentration can hardly be reached just with systemic application [7]. It is well known that insufficient antibiotic concentrations foster the development of antibiotic resistance. Because of that and in view of the fact that antibiotic-resistant bacteria is one of the biggest threats to global health, there is a pressing need for new therapeutic strategies.

Delivery of pharmaceutical agents directly to a desired location in the body using nanoparticles (NPs) is an approach, which aroused a lot of interest over the past decades. The unique and versatile characteristics of magnetic nanoparticles (MNPs) particularly iron oxide nanoparticles (IONPs) like small size, high surface to volume ratio, chemical stability, electromagnetic properties, biocompatibility and biodegradability [9–15] make them highly attractive for a lot of biomedical applications such as magnetic resonance imaging (MRI) [16], magnetic hyperthermia [17], targeted drug and gene delivery [18, 19], biosensing [20] and bioseperation [21] as well as many more [9–12]. The general underlying principle of the so-called drug targeting aims for an accumulation of a drug only in the desired tissue or interaction exclusively with targeted cells [22–24]. This is a desirable approach especially in targeted drug delivery for cancer treatment to avoid unwanted side effects on healthy cells [24] but it could also present a favorable approach to reach high local antibiotic concentrations on implants affected by bacterial biofilms [25]. To achieve an active targeting of drug carriers either a functionalization with target-cell specific structures such as antibodies or the guidance via an external functionality like a magnetic field is necessary. [10, 19, 22].

When foreign objects/materials such as nanoparticles enter the body, they are rapidly recognized by the host’s immune system. Nanoparticles are almost instantly victims of opsonization when administered intravenously leading to recognition of the opsonins by cells of the mononuclear phagocytic system (MPS) [26]. Accordingly, most of the nanoparticles are cleared from the circulation and get stuck in organs belonging to the reticuloendothelial system (RES) like liver and spleen before they can even reach their target [27]. Due to that fact, researchers focused on which characteristics of nanoparticles can influence this identification by the immune system and developed various strategies to overcome recognition by cells of the MPS like conjugation with polyethylene glycol (PEG) [28, 29], CD47 [30] or cloaking with membranes of blood cells [31, 32]. A recent in vivo study used PEGylated, magnetic silica nanoparticles and proved them as biocompatible in mice [33]. The fate of the nanoparticles after defined time points up to 42 days was tracked using fluorescence labeling. Another in vivo study evaluated the influence of macrophages on the systemic distribution by injecting clodronate liposomes [34], which are well known for their depleting effect on macrophages [35, 36]. This influence could be proved, but no information was gained about the fate of the nanoparticles in the time-span immediately after injection, since tracking via fluorescence was only able after time-consuming sample preparation.

Here, positron emission tomography (PET) followed by ex vivo biodistribution investigation using a gamma counter is  an alternative possibility. PET is a well-established nuclear imaging technique relying on the detection of a radiotracer/radioisotope [9, 37]. The combination of nuclear imaging techniques like PET or SPECT (single photon emission computed tomography) with computed tomography (CT) or magnetic resonance imaging (MRI) allows the fusion of functional and anatomical information, ensuring the precise localization of radioisotope accumulation [38–44]. For achieving the conjugation or internalization of these radionuclides onto or into new nanoparticles, today various bifunctional chelators (BFCs) are readily accessible. Consequently, the path of nanovesicles can be investigated visually, quantitatively, in real-time after their administration, offering unique advance over non-tracer-based imaging modalities. Dynamic PET and SPECT acquisition allows the registration of organ uptake values, ex vivo biodistribution, and pharmacokinetic parameters of labeled nanovesicles, including blood clearance and excretion times. Thus, although the method recommends special radiochemical apparatus and has obvious limitations (limited tracking time depending on selected radioisotope), hybrid PET/CT imaging systems combined with radiolabeled nanoparticles for targeted drug delivery offer a unique way to evaluate quantitatively the whole body distribution and the anatomical information regarding the NP-accumulation in targeted tissues [38, 44, 45].

**Fig. 1:**
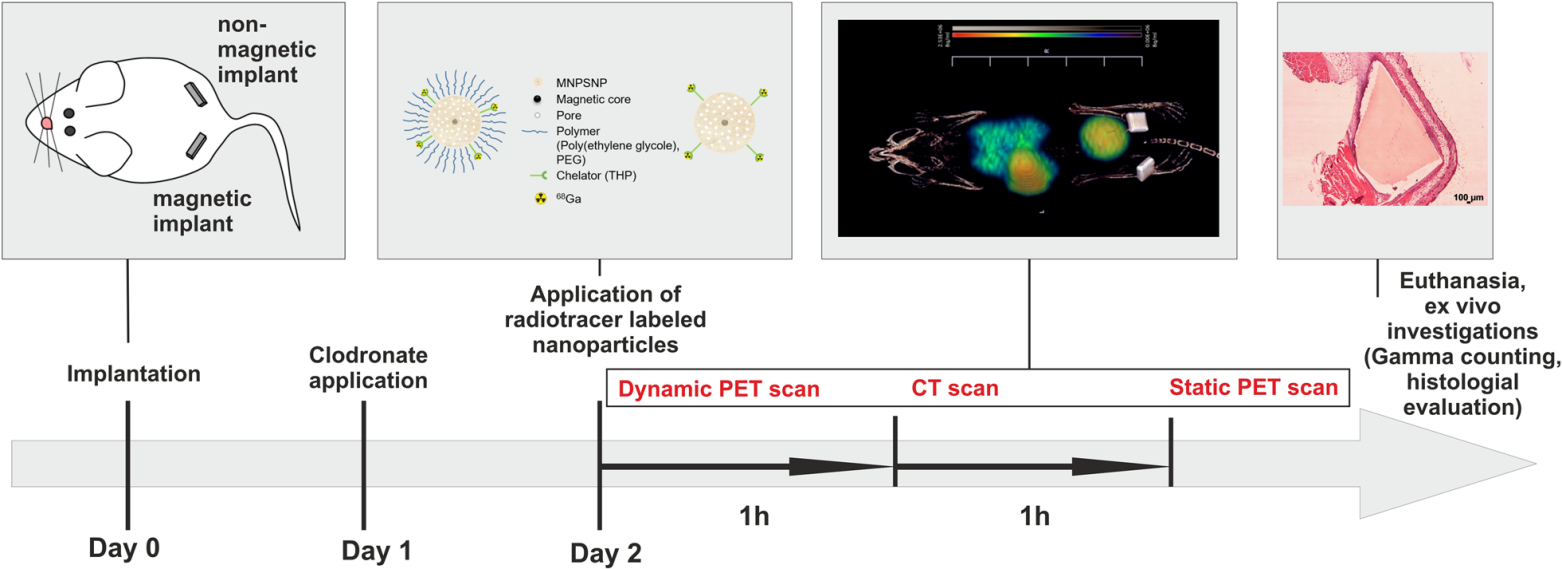
overview and order of events of the presented study

In the present study, two differently modified nanoparticles with a magnetic iron oxide core and a nanoporous silica shell (MNPSNP) radiolabeled with gallium-68 are under investigation in an in vivo mouse model. Radiolabeling was achieved by attaching the bifunctional chelator tris(hydroxypyridinone) (THP) for the first time onto such core–shell magnetic nanoparticles [46]. Additionally, to the nanoparticles, animals of some groups received a permanent magnet and a paramagnetic titanium control, which were implanted subcutaneously. Aim was to evaluate the effect of PEGylation and clodronate application on the early pharmacokinetics of the MNPSNP, and furthermore, to investigate their accumulation in organs and in the tissue surrounding a magnetic implant compared to a paramagnetic control (see Fig. 1).

## Results

### Synthesis of MNPSNP

**Fig. 2:**
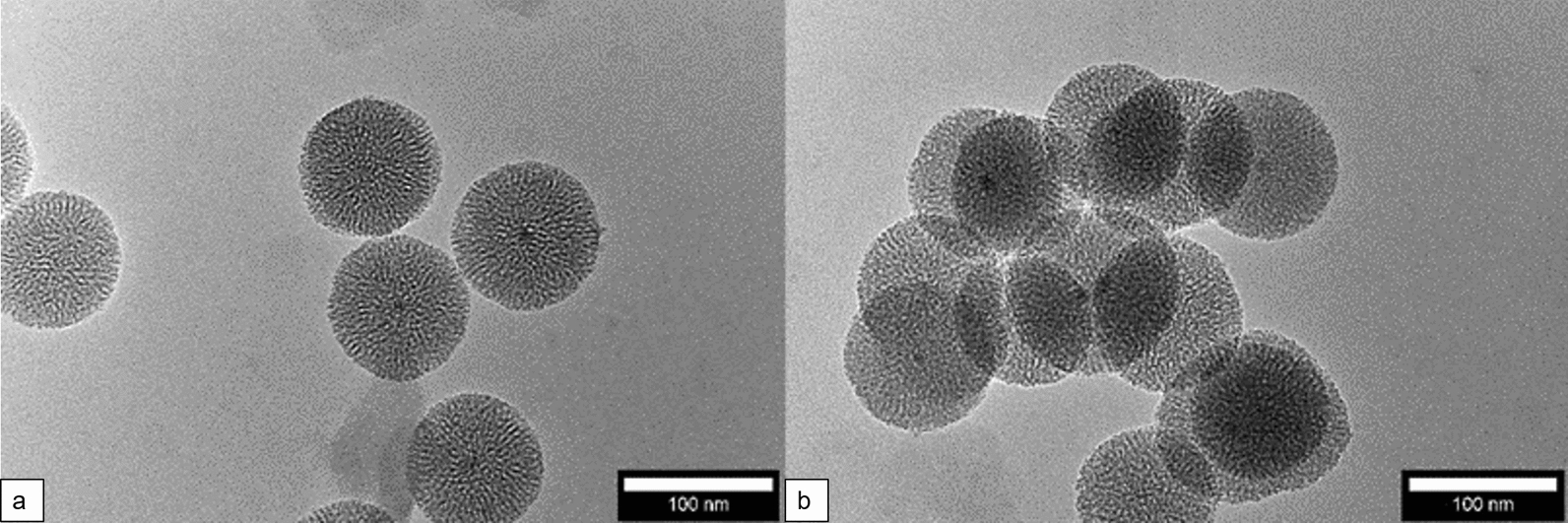
TEM images of MNPSNP@THP (**a**) and MNPSNP@THP-1kmPEG (**b**)

Synthesized MNPSNP have a size of 108 ± 9 nm as determined by TEM (transmission electronmicroscopy). After modification of the MNPSNP with THP (Tris(hydroxypyridinone)) and THP-1kmPEG (Polyethylene glycol) the appearance of the particles was preserved, i.e. similar diameter and no loss of the spherical shape (Fig. 2). Furthermore, the TEM investigations demonstrated the nanoporous structure within the shell of both types of nanoparticles.

The Fourier-transform infrared (FT-IR) spectra of the MNPSNP show the characteristic Fe–O stretching mode at 576 cm^−1^ as well as an intense Si–O–Si band at 1080 cm^−1^ [33]. Additionally, in the spectra of the THP-functionalized particles vibrations bands from 1709 to 1460 cm^−1^ are related to the vibration of carbonyl groups, amide and amines which are caused by the THP [47–49]. In the spectra of the THP-1kmPEG functionalized particles additional stretching modes of the methylene groups of the mPEG chain are present at 2980 to 2860 cm^−1^ [50].

**Fig. 3:**
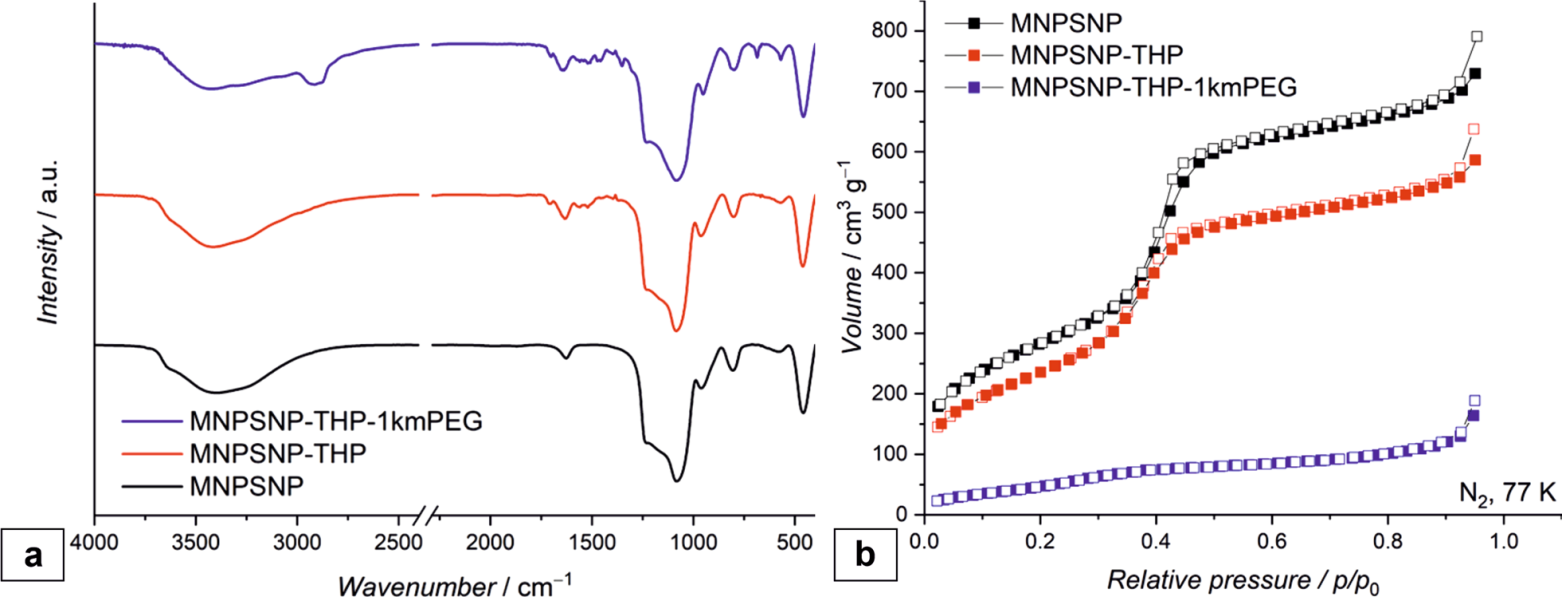
FT-IR spectra of unmodified MNPSNP (black), MNPSNP@THP (red) and MNPSNP@THP-1kmPEG (blue) (**a**); Nitrogen physisorption isotherms of unmodified MNPSNP, MNPSNP@THP and MNPSNP@THP-1kmPEG (**b**), filled squares: adsorption and empty squares desorption isotherm

The nitrogen physisorption (Fig. 3) was used to calculate the BET surface area, pore diameter and pore volume of the particles. The unmodified MNPSNP shows a high porosity with a BET surface area of 1021 m^2^ g^−1^, a pore diameter of 4.4 nm and a pore volume of 1.1 cm^3^ g^−1^. After the modification these parameters decreased, caused by the covalent attachment of THP and mPEG. Resulting in a BET surface area of 886 m^2^ g^−1^, a pore diameter of 4.2 nm and a pore volume of 0.9 cm^3^ g^−1^ for the THP functionalized particles. If additionally 1kmPEG is attached to the surface, a BET surface area of 214 m^2^ g^−1^, a pore diameter of 3.5 nm and a pore volume of 0.2 cm^3^ g^−1^ is obtained.

The functionalization could furthermore be verified by TGA (thermogravimetric analysis). Here, the THP-functionalized MNPSNP show a weight loss of 13% and the THP-1kmPEG functionalized MNPSNP show a weight loss of 38% which indicates the presence of a different amount of organic residues on the MNPSNP. Since the same amount of THP was used in each synthesis, the remaining organic residues for the MNSNP@THP-1kmPEG are attributed to the mPEG functionalization. Compared to this, the weight loss of unmodified MNPSNP was 7% due to the evaporation of adsorbed water and the condensation of surface silanol groups.

**Fig. 4:**
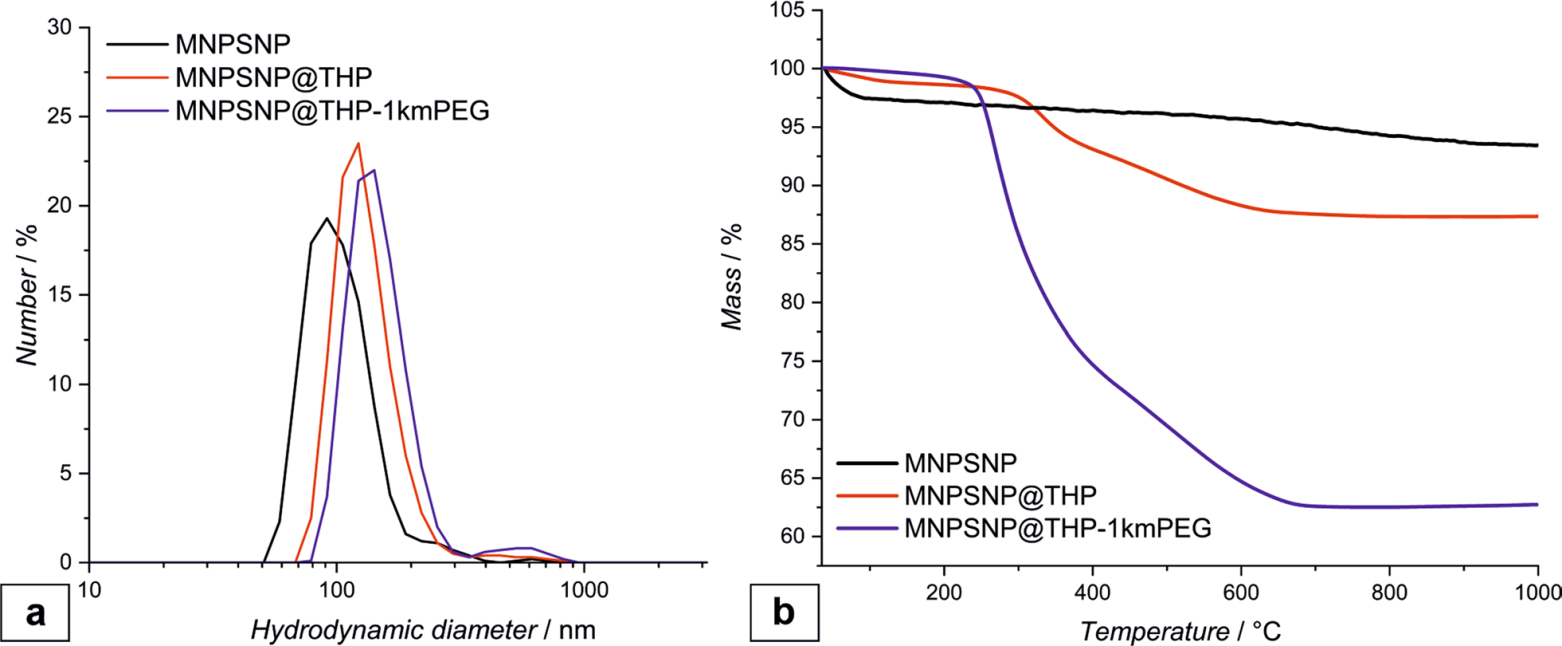
DLS measurement of unmodified MNPSNP (black), MNPSNP@THP (red) and MNPSNP@THP-1kmPEG (blue) (**a**); TGA curves of unmodified MNPSNP, MNPSNP@THP and MNPSNP@THP-1kmPEG (**b**)

The hydrodynamic diameter and the zeta-potential of the MNPSNP were measured using dynamic light scattering (DLS) method (Fig. 4). Unmodified MNPSNP showed a hydrodynamic diameter of 110 nm ± 20 nm and a zeta potential of − 29.0 mV ± 0.8 mV. After modification with THP the hydrodynamic diameter increased to 139 nm ± 7 nm, due to the larger hydration layer in aqueous media. Also, the zeta potential increased to – 22.3 mV ± 1.0 mV. Similar results were obtained with the THP-1kmPEG modification. Here, the hydrodynamic diameter increased to 163 nm ± 16 nm and the zeta-potential to 17.7 mV ± 0.4 mV. The higher increase in size and zeta potential is hereby caused by the PEGylation. Also, a slight aggregation of the MNPSNP@THP-1kmPEG was observable.

### ^68^Ga-radiolabeling of MNPSNP

**Fig. 5:**
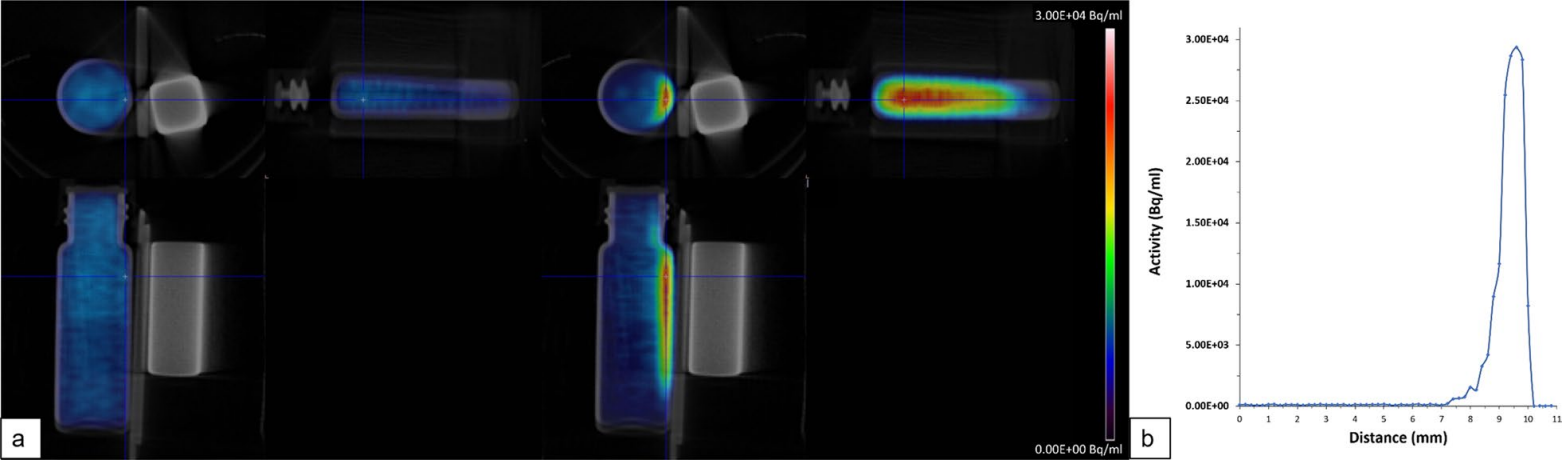
PET/CT images of the distribution of 5.3 MBq activity [^68^Ga]MNPSNP@THP in an ampoule exposed to neodymium magnet at the start of the 30 min dynamic acquisition and at 30 min (**a**) and central cross-section activity gradient in the vial at 30 min (**b**)

TLC and magnetic separation analysis revealed that both the MNPSNP@THP and MNPSNP@THP-1kmPEG variants rapidly complexed the ^68^Ga under continuous 600 RPM stirring. In each case, labeling efficiency reached min. 98% in 10 min, then the ^68^Ga-conjugation stability proved above 99% at 30 min, 1 h, 2 h and 3 h post-labeling. Importantly, the THP-functionalization allowed room temperature ^68^Ga-chelating reaction in slightly acidic bufferation (pH 4). These mild labeling conditions could exclude the possible modifications on the surface and in the structure of the MNPSNP. Radiochemical purity and particle integrity results were visually and quantitatively reinforced by a simple phantom PET/CT experiment (Fig. 5). PET/CT scans show that radioactive [^68^Ga]MNPSNP@THP migrated towards the strong magnetic field. Visual and ROI (region of interest) analysis proved an asymmetric radioactivity accumulation at the magnet side of the vial rapidly from the first moment of exposition, until 98.2% of the total ^68^Ga-activity could be detected near the magnet surface.

The magnetic and TLC analyses predicted the labeled compounds’ proper in vitro ^68^Ga-complexing stability in blood sera, as the labeling efficiency analysis resulted in comparable values (above 95% at 30 min, 1 h and 3 h).

### Evaluation of in vivo pharmacokinetics by PET/CT imaging

**Fig. 6:**
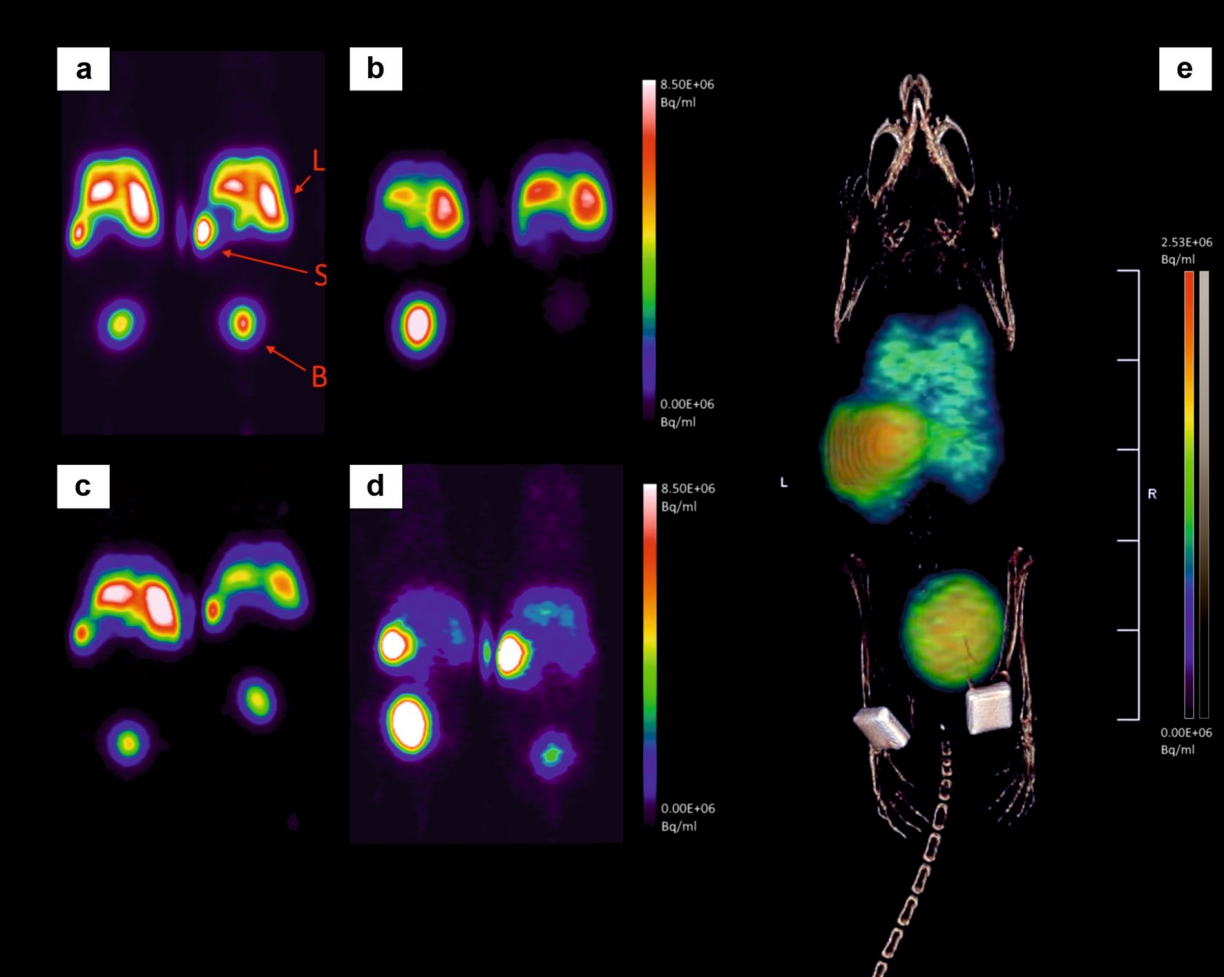
**a**–**d** Representative PET images of two mice from each group 60 min post-injection. **a** Group 1 + 3, b: Group 2 + 4, c: Group 5, d: Group 6. *L* liver, *S* spleen, *B* bladder. **e** representative fused PET/CT image (maximal intensity projection) of one mouse of Group 6 at 120 min p.i. Colored scale: coronal PET layer showing activity accumulation in the lung, liver, spleen and bladder; monochrome scale: CT layer with identifiable magnetic (left) and titanium (right) implants near the femora

Control groups without implantation and groups with implantation were combined for the PET/CT imaging study, resulting in four groups from the original six groups (Group 1 + 3: [^68^Ga]MNPSNP@THP without clodronate pretreatment, Group 2 + 4: [^68^Ga]MNPSNP@THP-1kmPEG without clodronate pre-treatment, Group 5: [^68^Ga]MNPSNP@THP with clodronate and Group 6: [^68^Ga]MNPSNP@THP-1kmPEG with clodronate). Implants are clearly recognizable in the fused PET/CT images by the CT therefore NP uptake on the implants or in the surrounding tissue could be measured (see Fig. 6).

**Fig. 7:**
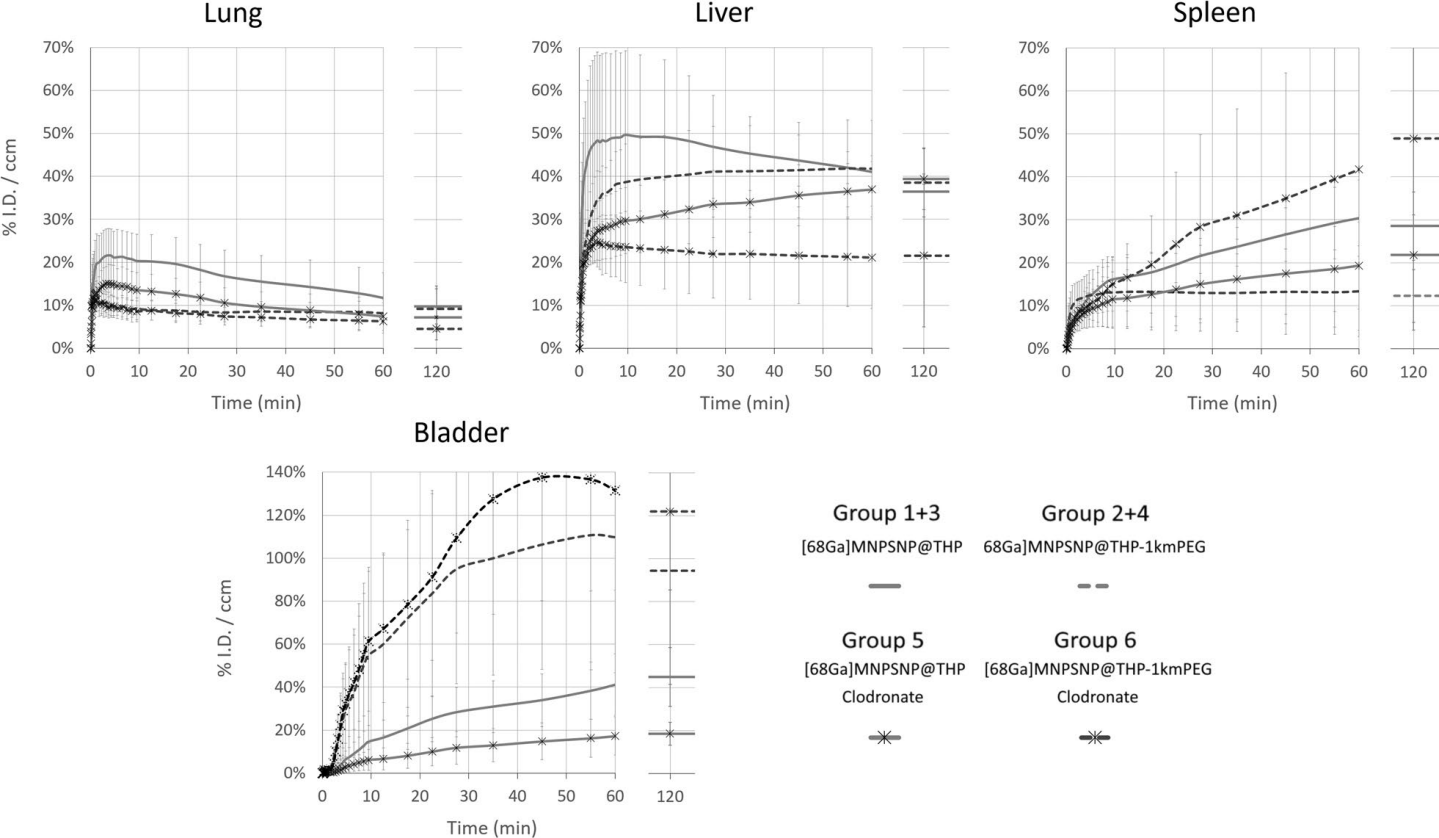
Lung, Liver, spleen and bladder time-activity curves (%ID/ml) of all groups, calculated from dynamic PET acquisition over 60 min and from static images at 120 min p.i. of [^68^Ga]MNPSNP@THP and [^68^Ga]MNPSNP@THP-1kmPEG

**Fig. 8:**
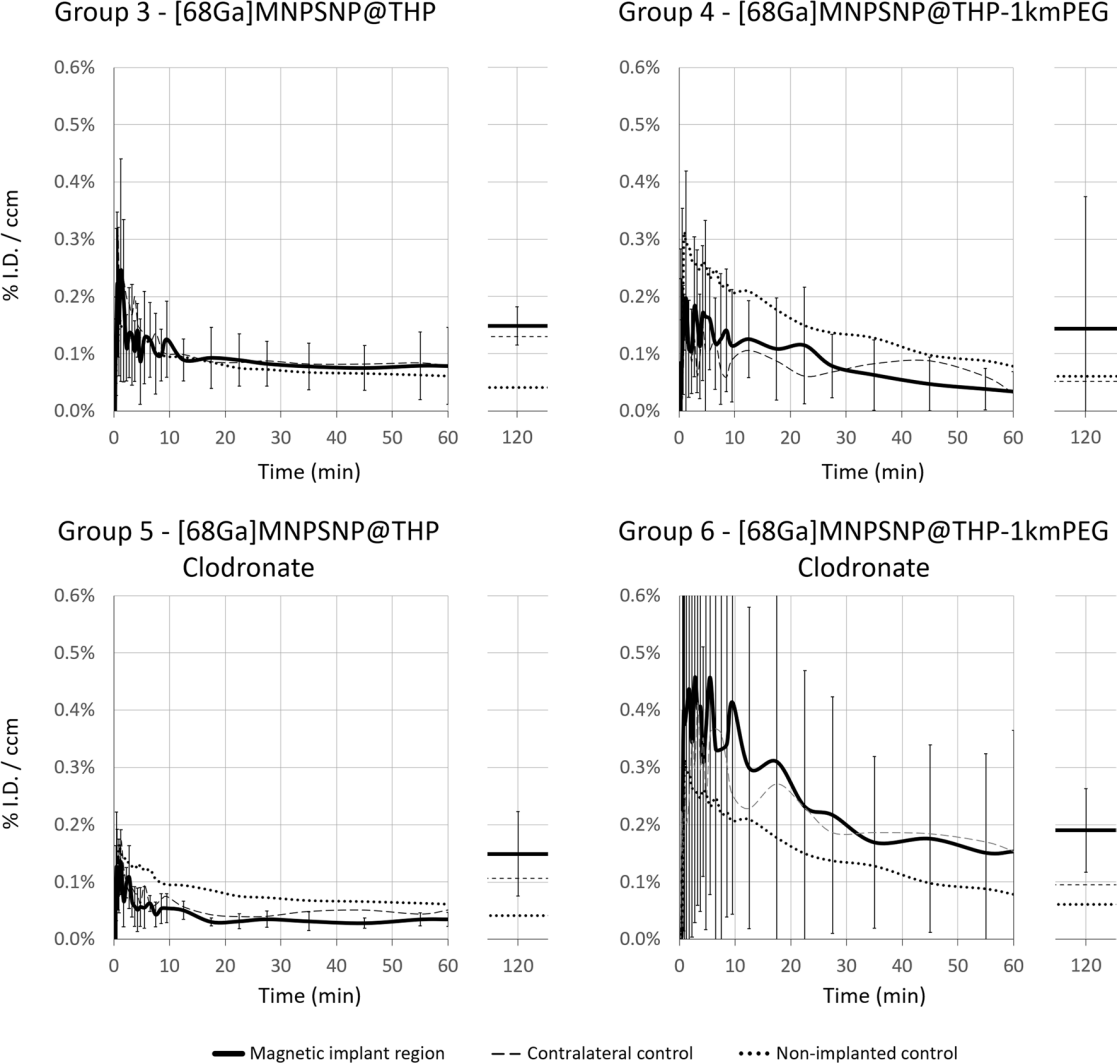
Implant region time-activity curves (%ID/ml), with contralateral control and non-implanted control, calculated from dynamic PET acquisition over 60 min and from static images at 120 min p.i. of [^68^Ga]MNPSNP@THP and [^68^Ga]MNPSNP@THP-1kmPEG

The pharmacokinetics and biodistribution of all radiolabeled nanoparticles could clearly be visualized and followed by 60 min dynamic PET/CT imaging in all animal groups. In general, both variants of ^68^Ga-labeled MNPSNP accumulated mainly in the RES organs (liver and spleen). Macrophage depletion and PEGylation had significant effects on the 60 min pharmacokinetic profiles both separately and in combination. Lung, liver, spleen and bladder uptakes were different and dependent on the nanoparticle variant and the clodronate pre-treatment (see discrete time-activity organ curves on Figs. 7 and 8 and detailed average %ID/ml ± SD values in Additional file 1: Table S1).

The highest lung uptake (avg. 21.60 ± 6.20% ID/ml) could be measured in the Group 1 + 3 ([^68^Ga]MNPSNP@THP, without clodronate). Faster kinetics/less binding in Group 2 + 4 indicate a difference between PEGylated and non-PEGylated NPs. Liver signal quickly accumulated in all animals, reaching a maximum liver uptake of avg. 49.66% ± 19.51% (SD) ID/ml at 9 min p.i. in the Group 1 + 3. PEGylation resulted in lower maximum liver uptakes as well as [^68^Ga]MNPSNP@THP-PEG administration combined with the clodronate treatment was able to further decrease this peak liver accumulation. Concurrently, a dynamically increasing uptake could be measured in the spleen reaching the highest values with an average %ID/ml of 41.68% ± 37.37% for Group 6 ([^68^Ga]MNPSNP@THP-1kmPEG, with clodronate) at 60 min p.i. and 48.91% ± 44.62% at 120 min. The urinary bladder activity had also strongly increased in the [^68^Ga]MNPSNP@THP-1kmPEG administered Group 2 + 4 and Group 6, concluding that the PEGylated NP variants had more rapidly migrated through the kidneys and urinary track. Implant uptakes remained below 1% ID/ml without distinct accumulation over time. The highest activity accumulation could be seen in Group 2 + 4 and Group 6 with avg %ID/ml of 0.22% ± 0.32% and 0.19% ± 0.07% at 120 min p.i., respectively.

The whole organ uptake estimations also revealed that the macrophage depletion had a substantial impact on RES organ uptake when [^68^Ga]MNPSNP@THP-1kmPEG were injected (see Additional file 1: Fig. S3 and Table S1). Lower whole lung and liver accumulation (62.4% ± 8.8% at 60 min p.i) could be estimated without clodronate injection as well, after [^68^Ga]MNPSNP@THP-1kmPEG were applied. Bladder activity was significantly higher (21.5% ± 9.5% at 60 min p.i, see p-values in Table 1).

**Table 1:** Significance (p-) values generated by comparison of the lung, liver (Liv), spleen (Spl) and bladder (Bla) %ID/ml activity concentration levels at 120 min using one-way ANOVA followed by Tukey or Games–Howell test

	Non-PEG (Group 1 + 3)		PEG (G2 + 4)		CL (G5)	
PEG + CL (Group 6)	Lung	0.083	Lung	0.135	Lung	0.714
	Liv	0.267	Liv	0.180	Liv	0.168
	Spl	0.699	Spl	0.299	Spl	0.520
	Bla	0.011	Bla	0.584	Bla	0.003
CL (Group 5)	Lung	0.678	Lung	0.808		
	Liv	0.740	Liv	0.997		
	Spl	0.536	Spl	0.264		
	Bla	< 0.001	Bla	0.002		
PEG (Group 2 + 4)	Lung	0.991				
	Liv	0.735				
	Spl	< 0.001				
	Bla	0.360				

Whole organ uptake estimations also revealed that clodronate injection combined with PEGylation resulted in dramatically decreased liver uptakes to 41.6%ID ± 19.6% at 60 min p.i., with slightly higher spleen accumulation (15.8% ± 12.4%).

Implant region uptakes remained below 0.5% I.D./ccm except for Group 6 in which slightly higher accumulation was observable in the nonmagnetic and magnetic implant regions (avg. 0.46% I.D./ccm at 2 and 5 min p.i., max. 1.00–1.37% I.D./ccm at 1–6 min p.i.) compared to the non-implanted tissue control. After the 60-min dynamic phase, slightly higher values could be measured in all groups at 120 min.

### Gamma counting

**Fig. 9:**
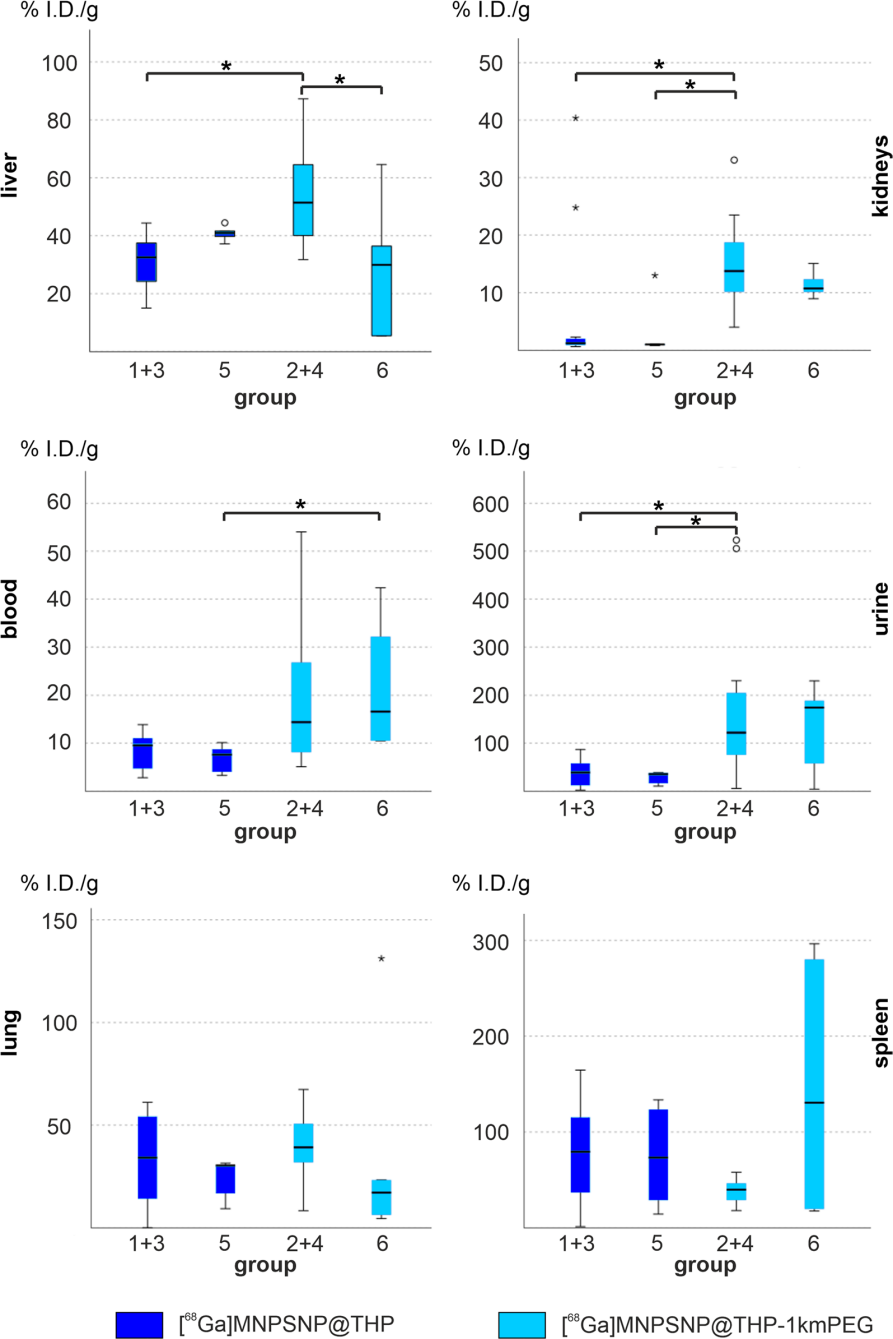
box-and-whisker plots of %I.D./g tissue of liver, kidney, blood, urine, lung, and spleen per group. The boxes represent the 25th to 75th percentiles, the black solid line indicates the median. Dark-blue: non-PEGylated groups; light-blue: PEGylated groups. Statistical significances are marked with asterisks *p < 0.05, **p < 0.01. Group 1 + 3 [^68^Ga]MNPSNP@THP, Group 2 + 4 [^68^Ga]MNPSNP@THP-1kmPEG, group 5 [^68^Ga]MNPSNP@THP and clodronate pretreatment, group 6 [^68^Ga]MNPSNP@THP-1kmPEG and clodronate pretreatment

The gamma counting results are expressed in % injected dose per gram tissue (%ID/g), which describes the activity concentration of the examined tissue or fluid as the half-life corrected percentage of the ID at the beginning of the dynamic scan referred to 1 gram. Comparing the four different groups, no significant differences could be found for organ values of the lungs (p > 0.05) and the spleen (p > 0.05). Mice which received [^68^Ga]MNPSNP@THP-1kmPEG showed significantly higher activity levels in the liver than mice receiving the same MNPSNP and additional clodronate injection (group 2 + 4 > group 6, p = 0.037) as well as mice which received [^68^Ga]MNPSNP@THP (group 2 + 4 > group 1 + 3, p = 0.01). Activity values in kidneys were significantly higher for the [^68^Ga]MNPSNP@THP-1kmPEG compared to the [^68^Ga]MNPSNP@THP (group 1 + 3 < group 2 + 4, p = 0.013). After clodronate pretreatment, the same trend was observed (group 5 < group 2 + 4, p = 0.035). When [^68^Ga]MNPSNP@THP-1kmPEG were administered, higher activity values for blood and urine could be measured with significant differences between groups with clodronate pretreatment for the blood (group 6 > group 5, p = 0.046). Activity values for urine of animals which received [^68^Ga]MNPSNP@THP-1kmPEG were significantly higher when compared to the values of animals which received [^68^Ga]MNPSNP@THP (group 2 + 4 > group 1 + 3, p = 0.017, group 2 + 4 > group 5, p = 0.038) (see Fig. 9).

**Fig. 10:**
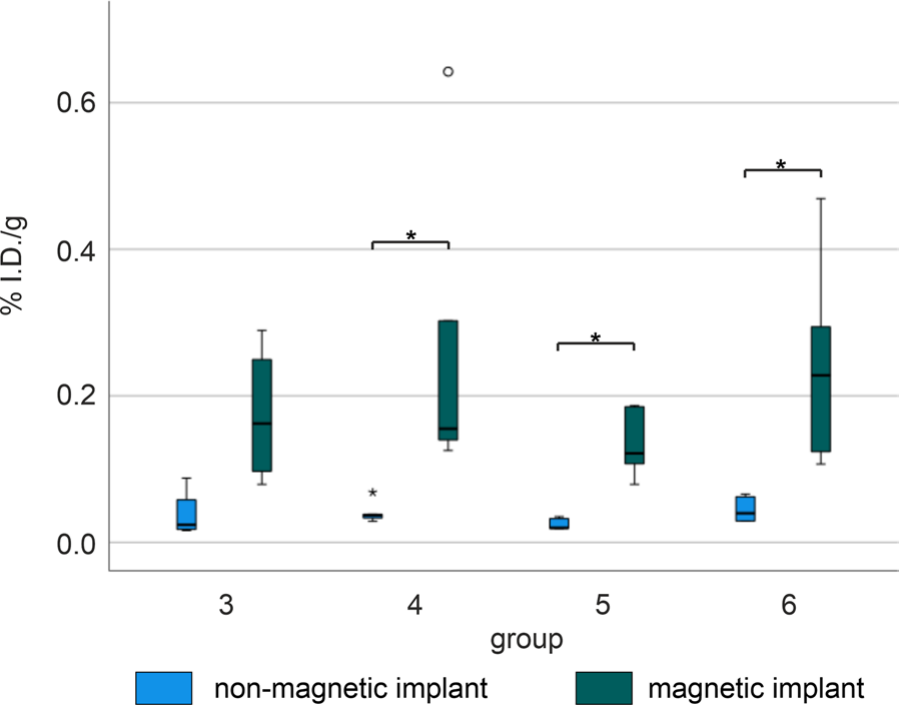
box and whisker plots of the %I.D./g tissue for the tissue surrounding the implants for the magnet (green) and titanium sides (blue) per group. The boxes represent the 25th to 75th percentiles, the black solid line indicates the median. Statistical significances are marked with asterisks *p < 0.05. group 3 [^68^Ga]MNPSNP@THPG, group 4 [^68^Ga]MNPSNP@THP-1kmPEG, group 5 [^68^Ga]MNPSNP@THP and clodronate pretreatment, group 6 [^68^Ga]MNPSNP@THP-1kmPEG and clodronate pretreatment

Taken as a whole, the %I.D./g of the samples of the tissue surrounding the implants are quite low compared to the values of the organs and collected body fluids. Considering group 4 to 6, all six samples could be used for analysis whereas in group 3 only four samples were available. Altogether, the activity was significantly higher in the samples of the magnetic implant compared to the para-magnetic titanium implant (control) for all groups except for group 3 (group 3 p = 0.068, group 4 p = 0.028, group 5 p = 0.043, group 6 p = 0.028, Fig. 10).

### In vivo metabolite study 

In the in vivo metabolite study, the fraction of free [^68^Ga]Ga3+ detected by radio-TLC for liver, lung, spleen, blood and urine was 96.9% ± 3.1%, with no significant difference between the tissues or body fluids, neither was a difference observed between the [^68^Ga]MNPSNP@THP or [^68^Ga]MNPSNP@THP-1kmPEG group. PET/CT scans of the homogenates in a magnetic field revealed no distinct activity accumulation or gradient towards the magnets (see Additional file 1: Fig. S4).

### Histological evaluation

**Fig. 11:**
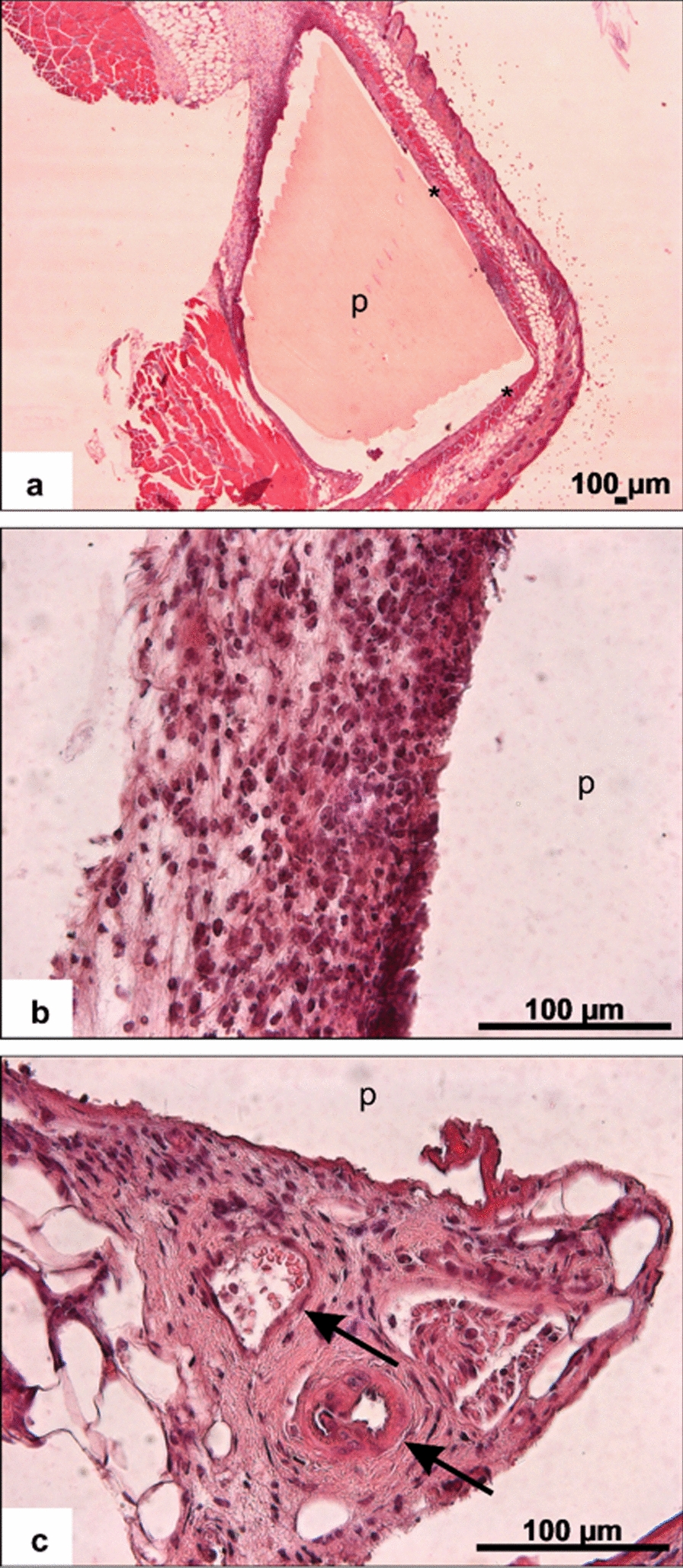
**a–c** H.E. staining of the tissue which surrounded the implant with former implant location (p) showing thin fibrous capsule (**a**,*) cell infiltration (**b**) and small vessels close to the former implantation site (**c**, black arrows)

The inserted placeholders were easily sliceable with the microtome. Sometimes they detached during the staining procedure but allowed in most cases an easy identification of the implantation site (Fig. 11a). Examination of the slices showed a thin fibrous capsule (Fig. 11a*) around the former implantation site in almost all slices investigated. Infiltration with immune cells, predominantly lymphocytes (Fig. 11b), was detectable in all samples to a varying degree. However, no group- or side-specific trends could be identified. In some samples, small vessels could be seen close to the prior implantation site (Fig. 11c).

## Discussion

To deliver drugs and other cargos to a specific area in the body via nanoparticles is a promising approach not only in cancer therapy, but it could also help overcome the limitations of antibiotic therapy of PJI. Especially, superparamagnetic nanoparticles consisting of an iron core encapsulated into a nanoporous silica shell are promising carriers for such applications. Their superparamagnetic properties allow targeting via a magnetic field either from an implant inside the body or from an externally applied magnetic field [12]. Although the general principle of magnetic drug targeting in the context of implant related topics was already proven [33, 34, 51], many details remain unclear and the delivery rate has to be enhanced.

Tracking of radiolabeled nanoparticles in a mouse model by PET/CT imaging will provide a better understanding of nanoparticle delivery to magnetic implants. The radionuclide ^68^Ga represents a cost-effective and ideal PET tracking tool for quantitative and non-invasive evaluation via PET/CT imaging. Moreover, it allows real-time visualization of the most critical first minutes to hours of such drug delivery systems in vivo. For the first time, the ^68^Ga-labeling of MNPSNP via the bifunctional THP-chelator proved successful. It offers rapid, simple and stable labeling, which enables 2–3 hours relevant PET tracking time [39]. In the current work, this method verified that stealth nanoparticles combined with macrophage depletion can nearly half the unwanted liver uptake of a drug delivery system. The ex vivo biodistribution study revealed accumulated activity for all organs investigated, and confirmed highest %I.D./g values for liver and spleen. No activity was detected on the blank implants themselves but in the tissue surrounding the implants. The accumulation for the implantation site of the magnet was significantly higher compared to the titanium control, proving that the magnetic implant is able to retain significantly more MNPSNP. However, in comparison with the inner organs, the overall amount was very low.

The general principle of targeted drug delivery of magnetic nanoparticles to a magnetic implant was already proven in different studies [34, 52]. In one of them, iron oxide nanoparticles loaded with fluorouracil (5-Fu) where used to show that a magnetic scaffold implanted subcutaneously is able to attract nanoparticles injected into the muscles surrounding the implantation site [52]. Nevertheless, local application of nanoparticles is only partly comparable with the study presented here, where particles were injected intravenously, and with that had to face many more biological barriers on their way to the implants. Anyhow, these studies are two of the few showing that an external magnetic field is not necessarily needed to guide nanoparticles to an implantation site.

The finding that only a very small amount of nanoparticles is able to reach the tissue surrounding the implants depicts one of the major limitations regarding nanomedicine: the predominant amount of nanoparticles is lost in the first pass in organs like liver, spleen or lungs [23] which is consistent with the gamma counting and dynamic PET/CT scan results in the present study. Some authors already attest nanomedicine a severe delivery problem [53, 54]. The highest uptake of nanoparticles in liver and spleen in the here presented study fits into the findings of many previous investigations. A study using a similar approach with ex vivo biodistribution via gamma counting also showed a distribution throughout the organs with highest %I.D./g tissue for liver and spleen. Iron oxide nanoparticles labeled with ^68^Ga were used as well, but a different coating was applied [39]. These organs as part of the RES system show high amounts of macrophages responsible for rapid recognition and capturing of nanoparticles.

Therefore, an enormous effort is put in investigating the processes mediating nanoparticle interaction with body fluids and cells. Various literature describes that after intravenous injection nanoparticles rapidly interact with diverse blood components like immunoglobulins, blood-clotting factors, and complement proteins, forming a so-called protein corona [27, 55–61]. The so built corona can be subdivided in a hard corona formed by proteins with strong binding affinity to the nanoparticles’ surface like immunoglobulins and apolipoproteins and the soft corona, which builds the outer layer of the corona, and is dominated by weakly bound proteins for example albumin [59, 60, 62, 63]. The whole process is also referred to as opsonization that only then enables recognition by and interaction with phagocytic cells [26, 27, 55, 64]. Probably this is exactly what happened to the nanoparticles used in this study and explains the rapid accumulation in liver and spleen. However, the significantly higher uptake of the nanoparticles at the magnetic implant is a clear proof-of-concept, and with a much longer circulation time one can assume a further increasing accumulation.

By implementing a range of methods like size-exclusion chromatography gel filtration, isothermal titration calorimetry and surface plasmon resonance studies, Cedervall et al*.* gained a deeper understanding of the nanoparticle protein interaction [65]. They found that protein adsorption is strongly dependent on the particle surface characteristics and size but on protein identity as well. Especially, surface hydrophobicity plays a major role [65]. A more recent study investigated the protein adsorption on different types of mesoporous silica nanoparticles (MSNs) using 10% and 100% fetal calf serum (FCS) focusing on the hard corona [66]. The most important finding of this study was that surface functionality, particle type, pore size, surface roughness and incubation time had a lesser influence on the extent of protein adsorption to the particles than the serum concentration. However, they could show that carboxyl-functionalization can decrease protein adsorption when compared to the all-silica MSNs. This indicates that surface functionalization can influence nanoparticle protein interactions.

In the here presented study, PEGylation was used as an approach to prolong circulation time and it was hypothesized that lower amounts of nanoparticles would be captured in RES organs resulting in increased amounts of nanoparticles at the implantation site. Grafting so-called stealth polymers to nanoparticle surfaces has become a favored approach to prolong the blood circulation time. Amongst various polymers, PEG has developed into kind of a gold standard in nanoparticle functionalization [55, 67–70]. Back in 1995, it was already shown that PEG is able to increase the circulation time of colloidal nanocarriers up to hours compared to only minutes for the non-functionalized counterparts [71]. A recent study showed that by using PEGylated liposomes the circulation time of the anticancer drug camptothecin can be drastically increased when compared to the free drug and a conventional liposomal formulation. The longest circulation time was achieved with a PEG molecular weight (MW) of 5000 Daltons (Da). Considering the organ uptake, a significant decrease in liver uptake could be shown for the two formulations with PEGylation compared to the conventional liposomal formulation of the drug [29]. In the here presented study, evaluation of the PET scans revealed a decrease in nanoparticle uptake in the RES organs for the [^68^Ga]MNPSNP@THP-1kmPEG nanoparticles, especially in combination with clodronate pretreatment. Regarding the accumulation of nanoparticles in the tissue surrounding the implants, no significant differences between the [^68^Ga]MNPSNP@THP-1kmPEG and [^68^Ga]MNPSNP@THP nanoparticles were present.

Different studies already investigated the influence of PEG molecular weight on the protein adsorption and circulation time of nanoparticles coming to varying results. Gref et al*.* demonstrated that an increase of the MW of PEG from 2000 to 5000 Da led to a severe decrease in protein adsorption but they could also show that a further increase was not beneficial [68]. In contrast to that, a study investigating the influence of PEG chain length and density on biodistribution in mice using PEG-grafted nanocapsules found that 20,000 Da PEG provided the longest circulation time and the lowest liver uptake [72]. The chain length of 1000 Da PEG in the presented study was used to minimize the enlargement of the particle diameter. However, the results indicate that PEGylation had a definite stealth effect compared to the non-PEGylated NP variant but it might not be sufficient to strongly prolong the circulation time and enhance the accumulation at the implants.

Although surface functionalization with polymers often yielded promising results [55, 67, 69, 70, 73], concerns about the safety of polymers were rising. Due to the fact that an accelerated blood clearance phenomenon can be seen after repeated injections of PEGylated nanocarriers, non- immunogenicity of PEG is questioned [74]. It is also discussed whether PEGylation may negatively influence nanoparticles’ performance as drug carriers because it hinders nanoparticle-cell interactions [67]. To further improve the half-life of nanoparticles in the bloodstream whilst avoiding PEGylation shortcomings some more biologically inspired strategies have been evolved, like coating with cell membranes [31, 32, 75, 76] or functionalization with CD47 [30, 55, 77, 78].

Although PEGylation and concurrent depletion of macrophages by clodronate injection decreased the amount of retained nanoparticles in the liver to nearly the half, this effect was recognizable neither in the spleen nor at the implants. A study investigating the effect of active targeting and macrophage depletion with clodronate liposomes on the tumor uptake of zirconium-89 labeled gold nanoparticles also found that in macrophage-depleted mice the liver uptake was significantly reduced but the spleen uptake was significantly higher [79]. They tried to explain this phenomenon with a faster recovery of splenic macrophages compared to the recovery of Kupffer cells in the liver, but also stated that further research is needed [79]. In another study slices of liver and spleen stained with rat antimouse F4/80 mAb were investigated on different time points after the injection of clodronate liposomes. Kupffer cells were counted and a complete depletion was shown on day two and three after the injection, reappearance started at day five. In contrast to that no apparent changes were found in the splenic macrophages. These findings lead them to the conclusion that macrophage depletion induced by clodronate liposomes seems to be restricted mainly to the liver [80]. The findings of these two studies match with the results of the PET evaluations presented here.

A rather unexpected finding of this study was the rapid accumulation of activity within the urine, which indicates a possible filtration of nanoparticles or smaller fractions of them in the kidneys, generally indicating a partly degradation or loss of the radiolabeled coating. The glomerular filtration barrier (GFB) of the kidney consists of three layers [81, 82], beginning with the endothelial cells that form the capillaries of the glomerulus, the glomerular basement membrane (GBM) and the podocytes. The GBM is a layer of extracellular matrix, which covers the sides of the glomeruli not facing the mesangium. The so called podocytes are specialized cells that engulf the glomerulus [82]. The combination of the different layers of the GFB prevents leakage of molecules larger than 6–8 nm into the urine [82]. With dimensions of 108 ± 9 nm the NPs used in this study should not be able to cross this barrier. Moreover, PEGylation of the nanoparticles and additional clodronate treatment seem to enhance the amount of activity found in the urine. Other studies also found that nanoparticles larger than 6–8 nm were able to cross over into the urine, which is summarized in a review by Adhipandito et al. [82] The main factors which contribute to renal filtration are size, charge, composition (organic vs. inorganic) and surface functionalization. However, they stated that the role of PEGylation on promoting renal clearance of large NPs is not clear yet [82]. In the set up used in this study it is also conceivable that the ^68^Ga-labeled PEG may have partly detached from the NPs resulting in gamma signals in the urine from fragmented particles rather than intact ^68^Ga-labeled MNPSNP. Evaluation of the labeling stability in vitro gave no reason to expect such separation after this period of time, but this is only partly comparable to the real in vivo situation. The in vivo metabolite study showed no loss of the single label (^68^Ga), but the applied analytics could only detect free [^68^Ga]Ga^3+^, but not discriminate between intact ^68^Ga-labeled MNPSNP and smaller ^68^Ga-labeled fragments of the particles. The missing activity gradients towards the magnets in the PET/CT scans of the homogenates in this in vivo metabolite study support the assumption of partly detached ^68^Ga-labeled fragments, and might be an explanation for the high activity accumulation found in urine.

In contrast to previous studies, that investigated biocompatibility and nanoparticle accumulation at later time points semi-quantitatively using fluorescence microscopy [33, 34], the focus of this study was to gain quantitative results regarding nanoparticle accumulation with special interest on the implantation site. Functionalization of these specific nanoparticles and labeling with a radioisotope for monitoring the biodistribution via PET/CT and gamma counting was a new approach. Considering this, a short investigation time interval was chosen to proof the feasibility of the method as well as the in vitro stability of the radiolabeling for this kind of nanoparticles. Furthermore, visualizing particle distribution instantly after i.v. injection was a major focus of the presented study. The here used radionuclide ^68^Ga can be obtained from a generator-based system which makes its easily available and provides a high proportion of positron decay (89%) of 1.9 MeV maximum energy [39]. Successful labeling of IONPs in various sizes and shapes with ^68^Ga is already described in the literature [39, 83, 84]. But it was the first time the bifunctional chelator THP was used for radiolabeling of such core–shell magnetic nanoparticles.

For possible following studies, changing the radioisotope to one with a longer half-life would allow tracing the nanoparticle´s fate over a longer period and might reveal possible accumulation at the implantation site at a later time point. In a study using the same location of the implants and the same type of nanoparticles an accumulation of nanoparticles at the implant after 24 h could be seen [34]. Zirconium-89 (^89^Zr) which is commonly used in clinical nuclear imaging, and due to its longer half-life of 78.4 h it allows longer tracking periods of up to several days [85]. This would allow repeated scanning of the animals at later time points without additional injections, which might reveal any later changes in particle distribution. Additionally, it can also be attached via a chelator molecule which might make it easy to transmit to the here presented nanoparticles [86]. Labeling nanoparticular systems with ^89^Zr is already described in the literature [85, 87]. However, as long as the blood circulation time of the nanoparticles is not increased it is questionable whether a longer study time would reveal any changes in nanoparticle distribution because a redistribution of particles caught in the RES organs is not likely to happen.

Using PET/CT and gamma counting to evaluate the accumulation of superparamagnetic nanoparticles in the region of a magnetic implant is a promising approach. Both methods revealed an accumulation of nanoparticles in the area of the implants, although only to a very small extent.

## Conclusions

Implant directed magnetic drug targeting using functionalized superparamagnetic core–shell silica nanoparticles  is a promising approach to treat implant infections in the future. Nevertheless, nanomedicine in general suffers from short blood circulation times and as a direct consequence low delivery rates at the desired area. The here presented study is no exception, as evidenced by the PET scan and gamma counting results, which showed high end values for liver and spleen. Especially because the PEGylation and clodronate injection used here did not provide satisfying results, further modifications of the nanoparticles e.g., with CD47 are necessary to prolong their overall blood circulation time and increase the at the moment very low amount of nanoparticles reaching the implantation site.

However, successful usage of nuclear imaging and analysis methods for implant-related questions enables future studies with differently modified nanoparticles, other implant materials or radioisotopes with a longer half-life to investigate other timeframes.

If it is possible to overcome this major limitation, nanoparticles carrying antibiotics could one day be successfully used for the treatment of implant infections.

## Materials and methods

### Materials

For the syntheses, all chemicals were used without further purification. Iron(II) chloride tetrahydrate (≥ 99%), iron(III) chloride tetrahydrate (99%), oleic acid (90%), chloroform (≥ 99%), cetyltrimethylammonium bromide (CTAB, ≥ 98%), ammonium hydroxide solution (≥ 25% NH_3_ in water), tetraethyl orthosilicate (TEOS, ≥ 99%) ethyl acetate (99.8%), (3-aminopropyl)triethoxysilane (APTES, 99%), acetonitrile (ACN, ≥ 99.9%) were purchased from Sigma-Aldrich Corporation (München, Germany). Poly(ethylene glycol) monomethylether (1kmPEG, M_w_: 1000 g/mol) was purchased from TCI Europe N.V. (Zwijindrecht, Belgium), tosylchloride (98%), dichlorodimethylsilane (98%) was purchased from abcr GmbH (Karlsruhe, Germany). Tetrahydrofuran (99.8%) was purchased from Acros Organics (Geel, Belgium), dichloromethane (DCM, ≥ 99.9%) was purchased from Honeywell GmbH (Seelze, Germany), MgSO_4_ (99%) was purchased from ThermoFisher (Kandel) GmbH (Kandel, Germany), tris(hydroxypyridinone)maleimide (THP-Mal) was purchased from CheMatech (Dijon, France). Silica gel impregnated glass fiber (ITLC-SG) chromatography plates were obtained from Merck (Darmstadt, Germany). The permanent block neodymium magnet that was used in the experiments was purchased from K&J Magnetics, Inc., USA (type: BX066, material: NdFeB, Grade N42, B_rmax_: 13,200 Gauss, B_Hmax_: 42 MGOe).

### Synthesis of MNPSNP

**Fig. 12:**
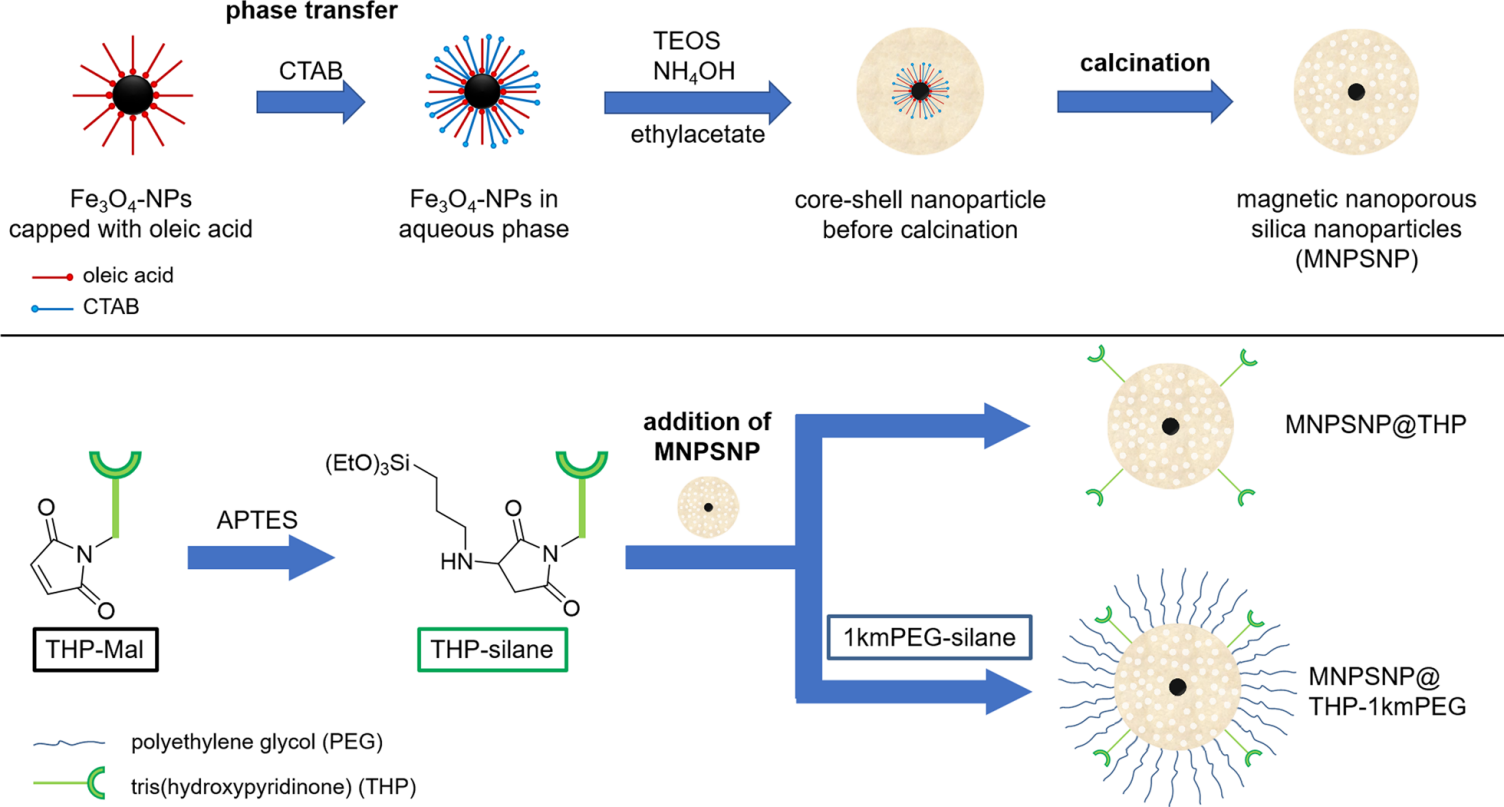
Synthesis and Functionalization of the MNPSNPs

The hydrophobic magnetite nanoparticles MNPSNP were prepared by a previously reported method with a slight modification.[33, 51] Briefly, the magnetite NPs are transferred into the aqueous phase using the surfactant CTAB. Next, water was added and the dispersion was stirred for 30 min at 60 °C. Afterwards ammonium hydroxide, TEOS and ethyl acetate are added to the reaction mixture and the reaction mixture was allowed to react for 3 h at 60 °C. After cooling, the light brown product was obtained after centrifugation, washed with ethanol and dryed under vacuum. The surfactant was then removed by calcination at 550 °C for 5 h with a heating rate of 1 °C/min (see Fig. 12).

### Synthesis of 1kmPEG-silane

The synthesis of 1kmPEG-silane was carried out using a two-step synthesis following a published procedure with some minor changes [69]. First, the tosylation of the 1kmPEG was achieved using tosylchloride in aqueous NaOH/THF mixture. The product was extracted three times with 50 ml DCM and the organic phase was dried using MgSO_4_. The solvent was removed by rotary evaporation and finally dried under high vacuum. The crude product (6.78 g) was used in the next step without further purification. It was refluxed with 1.37 ml APTES in 25 ml of chloroform for 17 h. After solvent removal and drying under vacuum, the product was stored as a 50 wt% solution in ethanol at 4 °C.

### Conjugation of THP and mPEG

For the conjugation of THP and 1kmPEG to the MNPSNP surface, the reaction flask was silylated using a mixture of 20 vol% dichlorodimethylsilane and 80 vol% chloroform. After cleaning the reaction flask 19.5 mg THP-Mal were dissolved in 45 ml ACN then 5.1 µl ml APTES were added and the mixture was refluxed for 2 h at 85 °C under N_2_. Afterwards the reaction mixture was allowed to cool down to 50 °C and 150 mg of the MNPSNP were added and the reaction mixture was further stirred for 24 h. For the additional functionalization with 1kmPEG-silane, 0.632 ml of the 1kmPEG-silane solution were also added with the MNPSNP. After cooling, the MNPSNP were obtained by centrifugation and washed three times with ethanol. The final product was dried under vacuum. These particles are denoted as MNPSNP@THP and MNPSNP@THP-1kmPEG (see Fig. 12).

### Characterization of the nanoparticles

Transmission electron microscopy (TEM) was performed with a FEI Tencai G2 F20 TMP instrument (C_S_ = 2 mm, C_C_ = 2 mm) with a 200 kV field emission gun in bright-field mode. For preparation, 400-mesh carbon coated copper grids (Plano GmbH) were used. The samples were dispersed in ethanol, dropped onto the grid and dried. The particle size was determined using NIH ImageJ. Fourier-transform infrared spectroscopy (FT-IR) was measured after a KBr pellet was prepared using a Bruker Tensor 27 instrument in transmission. N_2_ physisorption was measured on a Quantachrome Autosorb-3 after outgassing the sample for 24 h at 110 °C. Surface areas, pore sizes and pore volumes were calculated with the ASiQwin software (version 2.0) from Quantachrome. Thermogravimetric analysis (TGA) was measured with a Netzsch STA 449 F5 Jupiter. The samples were heated to 1000 °C in an Al_2_O_3_ crucible with a rate of 5 °C/min in an atmosphere of N_2_ and O_2_ (80% N_2_, 20% O_2_). Hydrodynamic size and zeta potential were measured by dynamic light scattering (DLS) with the Zetasizer Nano ZSP (Malvern Panalytical), wherefore the sample was dispersed in ethanol.

### Radiolabeling of MNPSNP@THP and MNPSNP@THP-1kmPEG with gallium-68 (^68^Ga)

Prior to the labeling, the [^68^Ga]GaCl_3_ eluate (1.1 ml, 0.1 M HCl, activity: 60.0 MBq–300.0 MBq) was obtained from a ^68^Ge/^68^Ga-generator (GalliAd^©^, Ire Elit, Fleurus, Belgium). The pH of the [^68^Ga]GaCl_3_ was adjusted by 0.1 M NaOH and 0.5 M phosphate buffer to pH 4. 50 mg of dry MNPSNP@THP and MNPSNP@THP-1kmPEG samples were resuspended in water by 10 min ultrasonication and the nanoparticle suspensions were added to the buffered [^68^Ga]GaCl_3_ solution. Labeling was performed by stirring the reaction mixture for 10 min at room temperature. In vitro radiolabeling stability was measured in the original labeling solution and in blood serum using thin layer chromatography (TLC) and magnetic separation of the NPs at different times post-labeling (10 min, 30 min, 1 h, 2 h and 3 h in solution and 30 min, 1 h and 2 h in serum at RT). For magnetic NP sedimentation a permanent neodymium magnet was used. Labeling efficiency was determined by measuring the removed solvent (supernatant) and sediment activities. As thin layer, silica gel impregnated instant thin layer chromatography (ITLC-SG) strips (Merck, Darmstadt, Germany) were developed by 0.1 M (pH = 4) citrate buffer. Yield values were calculated using the activities of origin point (Rf = 0) and solvent front (Rf = 1). Particle size distributions and zeta potential values were also checked 24 h post-labeling by DLS, the labeled NP samples were stored at + 4 °C until this analysis.

### Evaluation of particle integrity and labeling stability by PET/CT

Magnetic properties and traceability (labeling stability) of the labeled nanoparticles were tested in a small animal PET/CT system (Inveon; Siemens Healthineers, Erlangen, Germany). 5.0 ± 0.5 MBq activity [^68^Ga]MNPSNP@THP suspensions containing 5,5 mg NPs were diluted with PBS to 4 ml in 14 × 45 mm size test vials. The vials were placed onto a PET/CT animal bed in a magnetic field of neodymium magnets (Additional file 1: Fig. S1). This experimental phantom setup was dynamically scanned by PET for twenty minutes and a subsequent CT scan for structural information was performed. Fused PET/CT reconstruction of the phantom setup and activity accumulations were calculated by positioning rectangular region of interest (ROI) selections to the central cross-section of the vial in the direction of the magnet using Inveon Research Workplace (IRW 2.0) software.

### Animal model and in vivo setup

In vivo experiments were authorized according to the German Animal Welfare Act (registration number: 21/3722) and performed in 36 female BALB/cJHanZtm mice with an average body weight (BW) of 22.9 ± 1.28 g. Mouse husbandry was organized in nonmagnetic cages with a 14 h/10 h-day/night cycle and free access to food and tap water.

Twenty-four of the mice received a magnetic implant subcutaneously in the lateral femoral region on the left and a titanium implant on the right hind leg. Twelve animals did not undergo surgery and served as healthy control animals. For the magnetic implants commercially available neodymium magnets (5 × 5 × 2 mm, Webcraft AG, Germany) were purchased and coated with a ~ 300 nm layer of titanium (Institute for Micro Production Technology, Leibniz University Hannover) to provide biocompatibility. Implants out of Ti90Al6V4 (GoodFellow, England) with the same dimensions served as controls.

The mice were subdivided in six groups (n = 6 each group, see Table 2) to evaluate the accumulation of the two different nanoparticle configurations [^68^Ga]MNPSNP@THP and [^68^Ga]MNPSNP@THP-1kmPEG on the implants and in the implant surroundings as well as organ accumulation and excretion as important aspects in nanoparticle availability in the blood stream. Additionally, the influence of a macrophage depletion (+ C) was examined.

**Table 2:** group assignment of mice

Group	Day 0: Implantation	Day 1: Clodronate application	Day 2: application of radiolabeled nanoparticles and PET/CT-scan
1: NP	–	–	NP
2: PEG-NP	–	–	PEG-NP
3: I-NP	I	–	NP
4: I-PEG-NP	I	–	PEG-NP
5: I-C-NP	I	C	NP
6: I-C-PEG-NP	I	C	PEG-NP

#### Surgical procedure

**Fig. 13:**
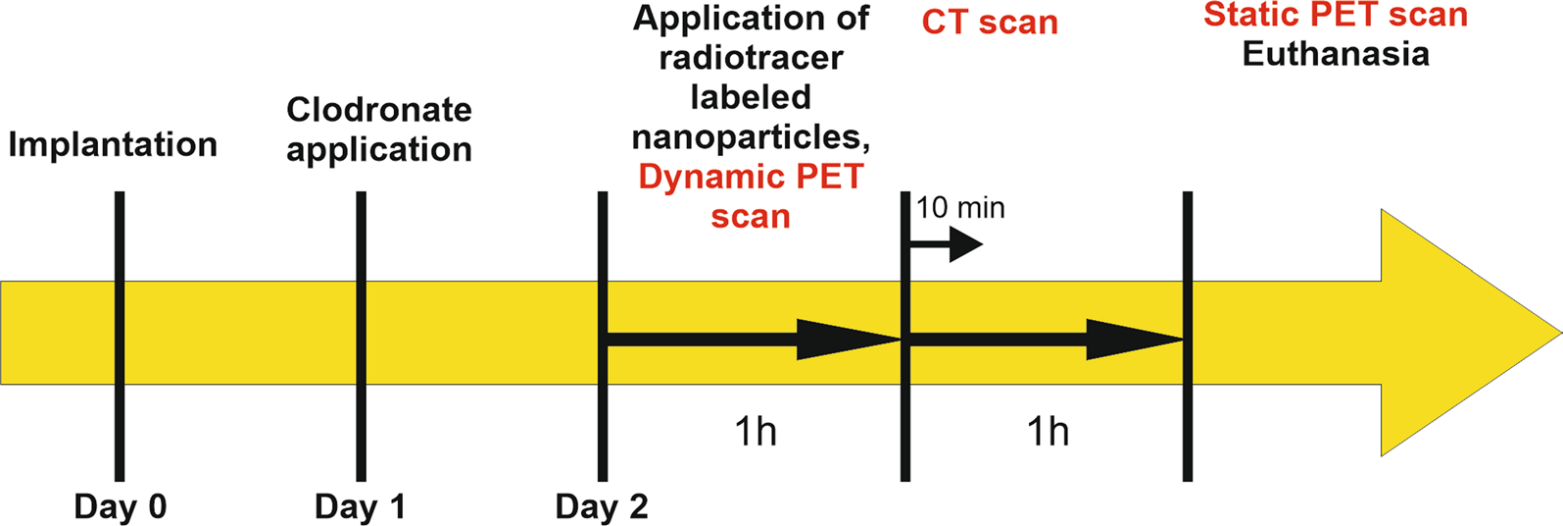
Timeline of the animal study

Surgery was performed under isoflurane anesthesia. Anesthesia was initiated in an inhalation chamber using 5% isoflurane in oxygen mixture. After unconsciousness occurred, an inhalation mask was applied and isoflurane concentration was reduced to 1–3% in oxygen mixture. Peri-operative analgesia was ensured by subcutaneous administration of carprofen. (Rimadyl^®^ 50 mg/ml, Zoetis, Germany) To avoid eye desiccation Bepanthen^®^ eye ointment was applied. The mice were put in prone position. After shaving, disinfection and local anesthesia with Prilocainhydrochlorid (Xylonest^®^ 0,5%, AstraZeneca GmbH, Germany), skin was incised approximately 7 mm central of the pelvis. In the meantime implants were prepared using a washing step with 70% ethanol (Emplura^®^, Merck KGaA, Germany) for at least 5 min and subsequent washing with sterile phosphate buffered saline (Dulbecco’s phosphate buffered saline, Biowest USA). Implants were inserted subcutaneously and moved slightly left (magnet) or right (titanium) in distal direction positioning them lateral to the femora whilst avoiding influences of the surgical sutures on the top of the implants and ensuring an adequate distance between the different implant materials. Each implant was fixed in place with a single suture Monocryl 6–0 (Ethicon, Germany) to avoid shifting. The skin incision was closed with two to three single button sutures with Monocryl 6–0. After surgery, mice received warmed isotone sodium chloride solution subcutaneously (0.0075 mg/g BW) and were placed under a heating lamp until recovery from anesthesia. During the postoperative follow-up, clinical examination was performed every day and carprofen was injected subcutaneously (0,005 mg/g BW) every twelve hours for pain relief. In the groups with macrophage depletion, mice received an intraperitoneal injection of 0.15 ml Clophosome^®^ (clodronate liposomes, approx. 1 mg/animal, FormuMax, USA) 24 h after implant placement (see Fig. 13).

#### PET/CT

The second postoperative day, nanoparticle application and PET/CT scans under isoflurane anesthesia were performed (see Fig. 13). Animals received a PBS buffered nanoparticle solution with a concentration of 5 µg/µl of either [^68^Ga]MNPSNP@THP or [^68^Ga]MNPSNP@THP-1kmPEG according to their experimental group. Applied activity in the animals varied between 5.0 MBq and 25.0 MBq in 100 µl saline, as the starting activity of the labeling procedure varied from 60.0 MBq to 300.0 MBq while the selected NP concentration remained 5 µg/µL. Mice were then subjected to serial PET imaging using a small animal PET/CT system (Inveon DPET and CT120; Siemens Healthineers, Erlangen, Germany). Dynamic PET images were acquired over a 60 min period from T_0_ after the i.v. administration. After that, 10-min low-dose CT scans were obtained to allow anatomic registration followed by static PET acquisition at 120 min p.i. over 10 min.

Fused PET/CT images were generated using Siemens Inveon Research Workplace (IRW 2.0) software. %ID/cm^3^ (%ID/ccm, %ID/ml) values (i.e. % injected activity percentage per unit volume organ) were determined from equal 3 mm diameter sphere shape 14 mm^3^ volume of interest (VOI) selections of the lungs, liver, spleen, urinary bladder and the implants on real time recorded PET images, guided by the CT layers. The dynamic pharmacokinetic curves were generated from these results. Lung, liver, spleen whole organ uptakes and whole urinary bladder activities were estimated by manual whole organ VOI selections.

After the second PET scan, mice were euthanized by cervical dislocation and intracardial blood sampling was performed. Spontaneously lost urine was collected whenever possible. In other cases, urine samples were taken by puncturing of the bladder. Implants were explanted and a placeholder was inserted instead to ensure an easier identification in later histological evaluation*.* The placeholders were fabricated out of polylactic acid (RAISE3D Premium PLA Filament, 1.7 mm, 3Dmensionals/Pontialis GmbH&Co. KG, Germany) with a geometry similar to the actual implants, using a 3D-printer (Raise3D Pro2 Plus 3D-printer with dual extruder, 3Dmensionals/Pontialis GmbH&Co. KG, Germany). Implants, surrounding tissue and organ samples (liver, spleen, kidney, and lung) were collected and weighed prior to fixation in 4% buffered formaldehyde solution.

#### In vivo metabolite study

In vivo metabolite experiments were authorized according to the German Animal Welfare Act (registration number: 20/3393) and performed in eight male BALB/cJ mice with an average body weight (BW) of 22.6 ± 0.7 g. Mouse husbandry was organized with a 14h/10h-day/night cycle and free access to food and tap water. Animals received a PBS buffered nanoparticle solution with a concentration of 5 µg/µl of either [^68^Ga]MNPSNP@THP or [^68^Ga]MNPSNP@THP-1kmPEG. Applied activity was in a range of 2.5 to 5.0 MBq in 100 µl saline in each animal. After an uptake phase of 30 min, mice were euthanized by cervical dislocation and intracardial blood sampling was performed. Samples of lung, liver, spleen, blood and urine were collected and diluted with PBS buffered saline to 1 ml. All samples were homogenized by ultrasound (Bandelin Sonoplus UW2070, booster horn SH70G, sonotrode MS73) for 1 min at 10% power (no pulse). Liver and spleen tissue required 2 min of ultrasound for complete homogenization. Small samples (1-5 µl) of the homogenates were analyzed by thin layer chromatography (ITLC-SG) strips using citrate buffer (0.1 M, pH = 4). Each set of vials (1.5 ml Eppendorf tubes) of the homogenates was placed in a holder together with permanent neodymium magnets (Additional file 1: Fig. S4). After 30 min, the holder was scanned by PET (10 min static scan) and CT. Fused PET/CT reconstruction of the homogenate vials were generated using the Inveon Research Workplace (IRW 2.0) software.

#### Gamma counting

Organ/fluid activities were counted in a 2470 WIZARD2 gamma counter from PerkinElmer (Waltham, MA, USA). %ID/g tissue results were calculated in MS Excel.

### Histological evaluation of tissue slices

The fixed samples of the tissue surrounding the implant with the placeholder included were dehydrated and paraffin-embedded. 5 µm thin slices were cut using a rotary microtome (Leica RM 2155, Leica Biosystems, Germany). Afterwards the slices were stained with hematoxylin–eosin and analyzed systematically using a light microscope (Axioskop 40 with AxioCam MRc digital camera and Zeiss AxioVision software, Carl Zeiss AG, Germany) beginning using the 25fold magnification to get a first overview and to identify the former implantation side. Afterwards, the 200 fold magnification was used to systematically investigate the tissue close to the implant for signs of inflammation or other structural changes. The different samples were compared with one another to detect any differences between the groups or between the sides with the different implants. Identification of nanoparticles themselves was not possible because no additional fluorescence functionalization of the MNPSNP was performed.

### Statistics

The data of 34 animals was used for final evaluation and statistical analysis. Two animals dropped out for evaluation, one due to terminal circulatory collapse during the CT scan and one due to sudden death the night after the clodronate injection. Statistical analysis was performed using SPSS 28 (IBM, USA). Tests were performed for the %I.D/g activity values of the organs (lung, liver, spleen, and kidney), blood, urine and for the tissue surrounding the implants (magnet, titanium) acquired by gamma counting (Kruskal–Wallis-Test). As well as for the activity levels generated from the static PET scan at 120 min of lung, liver, spleen and bladder acquired from the dynamic PET scan (one way ANOVA followed by Tukey or Games Howell test). If not stated otherwise, data are expressed as mean ± SD. Differences between the groups were considered statistically significant for p < 0.05.

### Supplementary Information

**Additional file 1: Figure S1.** Experimental phantom setup for testing the radiolabeled NPs in magnetic field by microPET/CT. **Figure S2.** Estimated whole organ uptake (%ID) levels of lung, liver, spleen and whole bladder (excreted) (A) and representative PET images (B) of two mice from each group, 60 min post-injection of ^68^Ga-labeled MNPSNP; L = liver, S = spleen, B = bladder. **Figure S3. **Estimated whole organ uptake (%ID) levels of lung, liver, spleen and whole bladder (excreted) during the 60 min dynamic PET acquisition. **Figure S4. **Experimental setup and PET/CT images of organ samples and body fluids for detection of *in vivo* metabolites in magnetic field by microPET/CT. Picture (A) and sketch (B) of the sample setup in the animal bed of the microPET/CT, and an exemplary microPET/CT image of samples of [^68^Ga]MNPSNP@THP-1kmPEG in healthy mice (C). **Table S1. **Estimated average whole organ uptakes (%ID) of lung, liver, spleen and whole bladder (excreted) during the 60 min dynamic PET acquisition. **Table S2.** Estimated whole organ uptake (%ID) levels of lung, liver, spleen and whole bladder (excreted) at 60 min and 120 min p.i.

## Data Availability

The datasets used and/or analyzed during the current study are available from the corresponding author on reasonable request.

## References

[1] Walter N, Rupp M, Hinterberger T, Alt V (2021). Prosthetic infections and the increasing importance of psychological comorbidities: an epidemiological analysis for Germany from 2009 through 2019. Orthopade.

[2] Kurtz SM, Lau E, Watson H, Schmier JK, Parvizi J (2012). Economic burden of periprosthetic joint infection in the united states. J Arthroplasty.

[3] Springer BD, Cahue S, Etkin CD, Lewallen DG, McGrory BJ (2017). Infection burden in total hip and knee arthroplasties: an international registry-based perspective. Arthroplast Today.

[4] Leta TH, Lygre SHL, Schrama JC, Hallan G, Gjertsen JE, Dale H (2019). Outcome of revision surgery for infection after total knee arthroplasty: results of 3 surgical strategies. JBJS Rev.

[5] Gundtoft PH, Overgaard S, Schonheyder HC, Moller JK, Kjærsgaard-Andersen P, Pedersen AB (2015). The “true” incidence of surgically treated deep prosthetic joint infection after 32,896 primary total hip arthroplasties. Acta Orthop.

[6] Rupp M, Walter N, Lau E, Worlicek M, Kurtz SM, Alt V (2021). Recent trends in revision knee arthroplasty in Germany. Sci Rep.

[7] Izakovicova P, Borens O, Trampuz A (2019). Periprosthetic joint infection: current concepts and outlook. EFORT Open Rev.

[8] Rimke C, Enz A, Bail HJ, Heppt P, Kladny B, von Lewinski G (2020). Evaluation of the standard procedure for the treatment of periprosthetic joint infections (PJI) in Germany—results of a survey within the EndoCert initiative. BMC Musculoskelet Disord.

[9] Vallabani NVS, Singh S, Karakoti AS (2018). Magnetic nanoparticles: current trends and future aspects in diagnostics and nanomedicine. Curr Drug Metab.

[10] Farzin A, Etesami SA, Quint J, Memic A, Tamayol A (2020). Magnetic nanoparticles in cancer therapy and diagnosis. Adv Healthc Mater.

[11] Elahi N, Rizwan M (2021). Progress and prospects of magnetic iron oxide nanoparticles in biomedical applications: a review. Artif Organs.

[12] Vangijzegem T, Stanicki D, Laurent S (2019). Magnetic iron oxide nanoparticles for drug delivery: applications and characteristics. Expert Opin Drug Deliv.

[13] Martins PM, Lima AC, Ribeiro S, Lanceros-Mendez S, Martins P (2021). Magnetic nanoparticles for biomedical applications: from the soul of the earth to the deep history of ourselves. ACS Appl Bio Mater.

[14] Ding Y, Zeng L, Xiao X, Chen T, Pan Y (2021). Multifunctional magnetic nanoagents for bioimaging and therapy. ACS Appl Bio Mater.

[15] Lamb J, Holland JP (2018). Advanced methods for radiolabeling multimodality nanomedicines for SPECT/MRI and PET/MRI. J Nucl Med.

[16] Weissleder R, Elizondo G, Wittenberg J, Rabito CA, Bengele HH, Josephson L (1990). Ultrasmall superparamagnetic iron oxide: characterization of a new class of contrast agents for MR imaging. Radiology.

[17] Pham HN, Pham THG, Nguyen DT, Phan QT, Le TTH, Ha PT (2017). Magnetic inductive heating of organs of mouse models treated by copolymer coated Fe3O4 nanoparticles. Adv Nat Sci Nanosci Nanotechnol..

[18] Yang L, Cao Z, Sajja HK, Mao H, Wang L, Geng H (2008). Development of receptor targeted magnetic iron oxide nanoparticles for efficient drug delivery and tumor imaging. J Biomed Nanotechnol.

[19] Liu S, Chen X, Bao L, Liu T, Yuan P, Yang X (2020). Treatment of infarcted heart tissue via the capture and local delivery of circulating exosomes through antibody-conjugated magnetic nanoparticles. Nat Biomed Eng.

[20] Liu Y, Li R, Zhang L, Guo S (2022). Nanomaterial-based immunocapture platforms for the recognition, isolation, and detection of circulating tumor cells. Front Bioeng Biotechnol.

[21] Schwaminger SP, Fraga-García P, Blank-Shim SA, Straub T, Haslbeck M, Muraca F (2019). Magnetic one-step purification of his-tagged protein by bare iron oxide nanoparticles. ACS Omega.

[22] Lübbe AS, Alexiou C, Bergemann C (2001). Clinical applications of magnetic drug targeting. J Surg Res.

[23] Bae YH, Park K (2011). Targeted drug delivery to tumors: myths, reality and possibility. J Control Release.

[24] Torchilin VP (2000). Drug targeting. Eur J Pharm Sci.

[25] Obermeier A, Küchler S, Matl FD, Pirzer T, Stemberger A, Mykhaylyk O (2012). Magnetic drug targeting as new therapeutic option for the treatment of biomaterial infections. J Biomater Sci Polym Ed.

[26] Gustafson HH, Holt-Casper D, Grainger DW, Ghandehari H (2015). Nanoparticle uptake: the phagocyte problem. Nano Today.

[27] Sousa De Almeida M, Susnik E, Drasler B, Taladriz-Blanco P, Petri-Fink A, Rothen-Rutishauser B (2021). Understanding nanoparticle endocytosis to improve targeting strategies in nanomedicine. Chem Soc Rev.

[28] Gal N, Lassenberger A, Herrero-Nogareda L, Scheberl A, Charwat V, Kasper C (2017). Interaction of size-tailored PEGylated iron oxide nanoparticles with lipid membranes and cells. ACS Biomater Sci Eng.

[29] Sivadasan D, Sultan MH, Madkhali OA, Alessa AA, Alsabei SH (2022). Stealth liposomes (PEGylated) containing an anticancer drug camptothecin: in vitro characterization and in vivo pharmacokinetic and tissue distribution study. Molecules.

[30] Jalil AR, Tobin MP, Discher DE (2022). Suppressing or enhancing macrophage engulfment through the use of CD47 and related peptides. Bioconjug Chem.

[31] Chen ZA, Wu SH, Chen P, Chen YP, Mou CY (2019). Critical features for mesoporous silica nanoparticles encapsulated into erythrocytes. ACS Appl Mater Interfaces.

[32] Piao JG, Wang L, Gao F, You YZ, Xiong Y, Yang L (2014). Erythrocyte membrane is an alternative coating to polyethylene glycol for prolonging the circulation lifetime of gold nanocages for photothermal therapy. ACS Nano.

[33] Janßen HC, Angrisani N, Kalies S, Hansmann F, Kietzmann M, Warwas DP (2020). Biodistribution, biocompatibility and targeted accumulation of magnetic nanoporous silica nanoparticles as drug carrier in orthopedics. J Nanobiotechnology..

[34] Reifenrath J, Janßen HC, Warwas DP, Kietzmann M, Behrens P, Willbold E (2020). Implant-based direction of magnetic nanoporous silica nanoparticles: influence of macrophage depletion and infection. Nanomedicine.

[35] Selander KS, Mönkkönen J, Karhukorpi EK, Härkönen P, Hannuniemi R, Väänänen HK (1996). Characteristics of clodronate-induced apoptosis in osteoclasts and macrophages. Mol Pharmacol.

[36] Kozicky LK, Sly LM (2019). Depletion and reconstitution of macrophages in mice. Methods Mol Biol.

[37] Milne S, King GG (2014). Advanced imaging in COPD: insights into pulmonary pathophysiology. J Thorac Dis.

[38] Polyak A, Ross TL (2018). Nanoparticles for SPECT and PET imaging: towards personalized medicine and theranostics. Curr Med Chem.

[39] Karageorgou MA, Vranješ-Djurić S, Radović M, Lyberopoulou A, Antić B, Rouchota M, et al. Gallium-68 labeled iron oxide nanoparticles coated with 2,3-dicarboxypropane-1,1-diphosphonic acid as a potential PET/MR imaging agent: a proof-of-concept study. Contrast Media Mol Imaging. 2017;2017: 6951240 10.1155/2017/6951240 PMC5763103 29445321

[40] Herzog H (2012). PET/MRI: challenges, solutions and perspectives. Z Med Phys.

[41] Judenhofer MS, Wehrl HF, Newport DF, Catana C, Siegel SB, Becker M (2008). Simultaneous PET-MRI: a new approach for functional and morphological imaging. Nat Med.

[42] Thomas G, Boudon J, Maurizi L, Moreau M, Walker P, Severin I (2019). Innovative magnetic nanoparticles for PET/MRI bimodal imaging. ACS Omega.

[43] Naszályi Nagy L, Polyak A, Mihály J, Szécsényi Á, Szigyártó IC, Czégény ZS (2016). Silica@zirconia@poly(malic acid) nanoparticles: promising nanocarriers for theranostic applications. J Mater Chem B.

[44] Polyak A, Naszalyi Nagy L, Mihaly J, Görres S, Wittneben A, Leiter I (2017). Preparation and (68)Ga-radiolabeling of porous zirconia nanoparticle platform for PET/CT-imaging guided drug delivery. J Pharm Biomed Anal.

[45] Polyak A, Képes Z, Trencsényi G (2023). Implant imaging: perspectives of nuclear imaging in implant, biomaterial, and stem cell research. Bioengineering.

[46] Santos MA, Gil M, Marques S, Gano L, Cantinho G, Chaves S (2002). N-carboxyalkyl derivatives of 3-hydroxy-4-pyridinones: synthesis, complexation with Fe(III), Al(III) and Ga(III) and in vivo evaluation. J Inorg Biochem.

[47] Faghihi K, Moghanian H (2010). Synthesis and characterization of new optically active poly(amide-imide)s containing 1,3,4-oxadiazole moiety in the main chain. Polym Bull.

[48] Paluszkiewicz C, Stodolak E, Hasik M, Blazewicz M (2011). FT-IR study of montmorillonite-chitosan nanocomposite materials. Spectrochim Acta A Mol Biomol Spectrosc.

[49] Zhao J, Wang J (2015). Understanding the amide-II vibrations in β-peptides. J Phys Chem B.

[50] Thibault-Starzyk F, Payen R, Lavalley JC (1996). IR evidence of zeolitic hydroxy insertion in amide formation by the Ritter reaction. Chem Commun.

[51] Janßen HC, Warwas DP, Dahlhaus D, Meißner J, Taptimthong P, Kietzmann M (2018). In vitro and in vivo accumulation of magnetic nanoporous silica nanoparticles on implant materials with different magnetic properties. J Nanobiotechnology.

[52] Ge J, Zhang Y, Dong Z, Jia J, Zhu J, Miao X (2017). Initiation of targeted nanodrug delivery in vivo by a multifunctional magnetic implant. ACS Appl Mater Interfaces.

[53] Wilhelm S, Tavares AJ, Dai Q, Ohta S, Audet J, Dvorak HF (2016). Analysis of nanoparticle delivery to tumours. Nat Rev Mater.

[54] Torrice M (2016). Does nanomedicine have a delivery problem?. ACS Cent Sci.

[55] Fam SY, Chee CF, Yong CY, Ho KL, Mariatulqabtiah AR, Tan WS (2020). Stealth coating of Nanoparticles in drug-delivery systems. Nanomaterials.

[56] Moghimi SM, Hunter AC, Murray JC (2001). Long-circulating and target-specific nanoparticles: theory to practice. Pharmacol Rev.

[57] Frank MM, Fries LF (1991). The role of complement in inflammation and phagocytosis. Immunol Today.

[58] Urban DA, Rodriguez-Lorenzo L, Balog S, Kinnear C, Rothen-Rutishauser B, Petri-Fink A (2016). Plasmonic nanoparticles and their characterization in physiological fluids. Colloids Surf B Biointerfaces.

[59] Gunawan C, Lim M, Marquis CP, Amal R (2014). Nanoparticle-protein corona complexes govern the biological fates and functions of nanoparticles. J Mater Chem B.

[60] Milani S, Baldelli Bombelli F, Pitek AS, Dawson KA, Rädler J (2012). Reversible versus irreversible binding of transferrin to polystyrene nanoparticles: soft and hard corona. ACS Nano.

[61] Gómez-Vallejo V, Puigivila M, Plaza-García S, Szczupak B, Piñol R, Murillo JL (2018). PEG-copolymer-coated iron oxide nanoparticles that avoid the reticuloendothelial system and act as kidney MRI contrast agents. Nanoscale.

[62] Lundqvist M, Stigler J, Elia G, Lynch I, Cedervall T, Dawson KA (2008). Nanoparticle size and surface properties determine the protein corona with possible implications for biological impacts. Proc Natl Acad Sci U S A.

[63] Lynch I, Dawson KA (2008). Protein-nanoparticle interactions. Nano Today.

[64] Liu T, Choi H, Zhou R, Chen IW (2015). RES blockade: a strategy for boosting efficiency of nanoparticle drug. Nano Today.

[65] Cedervall T, Lynch I, Lindman S, Berggård T, Thulin E, Nilsson H (2007). Understanding the nanoparticle-protein corona using methods to quntify exchange rates and affinities of proteins for nanoparticles. Proc Natl Acad Sci USA.

[66] Lin CY, Yang CM, Lindén M (2019). Influence of serum concentration and surface functionalization on the protein adsorption to mesoporous silica nanoparticles. RSC Adv.

[67] Amoozgar Z, Yeo Y (2012). Recent advances in stealth coating of nanoparticle drug delivery systems. WIREs Nanomed Nanobiotechnol.

[68] Gref R, Lück M, Quellec P, Marchand M, Dellacherie E, Harnisch S (2000). ‘Stealth’ corona-core nanoparticles surface modified by polyethylene glycol (PEG): influences of the corona (PEG chain length and surface density) and of the core composition on phagocytic uptake and plasma protein adsorption. Colloids Surf B Biointerfaces.

[69] Cauda V, Argyo C, Bein T (2010). Impact of different PEGylation patterns on the long-term bio-stability of colloidal mesoporous silica nanoparticles. J Mater Chem.

[70] Shim G, Miao W, Ko S, Park GT, Kim JY, Kim MG (2017). Immune-camouflaged graphene oxide nanosheets for negative regulation of phagocytosis by macrophages. J Mater Chem B.

[71] Verrecchia T, Spenlehauer G, Bazile DV, Murry-Brelier A, Archimbaud Y, Veillard M (1995). Non-stealth (poly(lactic acid/albumin)) and stealth (poly(lactic acid-polyethylene glycol)) nanoparticles as injectable drug carriers. J Control Release.

[72] Mosqueira VCF, Legrand P, Morgat JL, Vert M, Mysiakine E, Gref R (2001). Biodistribution of long-circulating PEG-grafted nanocapsules in mice: effects of PEG chain length and density. Pharm Res.

[73] Gref R, Lück M, Quellec P, Marchand M, Dellacherie E, Harnisch S (2000). ‘Stealth’ corona-core nanoparticles surface modified by polyethylene glycol (PEG). Colloids Surf B Biointerfaces.

[74] Ishida T, Kiwada H (2004). Accelerated blood clearance of pegylated liposomes after repeated injection. Drug Deliv Syst.

[75] Rao L, Xu JH, Cai B, Liu H, Li M, Jia Y (2016). Synthetic nanoparticles camouflaged with biomimetic erythrocyte membranes for reduced reticuloendothelial system uptake. Nanotechnology.

[76] Hu CM, Fang RH, Wang KC, Luk BT, Thamphiwatana S, Dehaini D (2015). Nanoparticle biointerfacing by platelet membrane cloaking. Nature.

[77] Gheibihayat SM, Jaafari MR, Hatamipour M, Sahebkar A (2021). Improvement of the pharmacokinetic characteristics of liposomal doxorubicin using CD47 biomimickry. J Pharm Pharmacol.

[78] Liu C, Yu D, Ge F, Yang L, Wang Q (2019). Fluorescent and mass spectrometric evaluation of the phagocytic internalization of a CD47-peptide modified drug-nanocarrier. Anal Bioanal Chem.

[79] Sobol NB, Korsen JA, Younes A, Edwards KJ, Lewis JS (2021). ImmunoPET imaging of pancreatic tumors with 89Zr-labeled gold nanoparticle-antibody conjugates. Mol Imaging Biol.

[80] Ohara Y, Oda T, Yamada K, Hashimoto S, Akashi Y, Miyamoto R (2012). Effective delivery of chemotherapeutic nanoparticles by depleting host Kupffer cells. Int J Cancer.

[81] Kamaly N, He JC, Ausiello DA, Farokhzad OC (2016). Nanomedicines for renal disease: current status and future applications. Nat Rev Nephrol.

[82] Adhipandito CF, Cheung SH, Lin YH, Wu SH (2021). Atypical renal clearance of nanoparticles larger than the kidney filtration threshold. Int J Mol Sci.

[83] Pellico J, Ruiz-Cabello J, Saiz-Alía M, del Rosario G, Caja S, Montoya M (2016). Fast synthesis and bioconjugation of 68Ga core-doped extremely small iron oxide nanoparticles for PET/MR imaging. Contrast Media Mol Imaging.

[84] Madru R, Tran TA, Axelsson J, Ingvar C, Bibic A, Ståhlberg F (2013). (68)Ga-labeled superparamagnetic iron oxide nanoparticles (SPIONs) for multi-modality PET/MR/Cherenkov luminescence imaging of sentinel lymph nodes. Am J Nucl Med Mol Imaging.

[85] Polyak A, Bankstahl JP, Besecke KFW, Hozsa C, Triebert W, Pannem RR (2021). Simplified 89Zr-labeling protocol of oxine (8-hydroxyquinoline) enabling prolonged tracking of liposome-based nanomedicines and cells. Pharmaceutics.

[86] Psimadas D, Georgoulias P, Valotassiou V, Loudos G (2012). Molecular nanomedicine towards cancer: 111In-labeled nanoparticles. J Pharm Sci.

[87] Starmans LWE, Hummelink MAPM, Rossin R, Kneepkens ECM, Lamerichs R, Donato K (2015). 89 Zr- and Fe-labeled polymeric micelles for dual modality PET and T 1 -weighted MR imaging. Adv Healthc Mater.

