# Supplementary material for: Preparation and PET/CT imaging of implant directed 68Ga-labeled magnetic nanoporous silica nanoparticles
Source: J Nanobiotechnology. 2023 Aug 17;21:270. doi: 10.1186/s12951-023-02041-8 (PMC10433681; doi:10.1186/s12951-023-02041-8)
Supplement: Supplementary file 1 — Additional file 1: Figure S1. Experimental phantom setup for testing the radiolabeled NPs in magnetic field by microPET/CT. Figure S2. Estimated whole organ uptake (%ID) levels of lung, liver, spleen and whole bladder (excreted) (A) and representative PET images (B) of two mice from each group, 60 min post-injection of 68Ga-labeled MNPSNP; L = liver, S = spleen, B = bladder. Figure S3. Estimated whole organ uptake (%ID) levels of lung, liver, spleen and whole bladder (excreted) during the 60 min dynamic PET acquisition. Figure S4. Experimental setup and PET/CT images of organ samples and body fluids for detection of in vivo metabolites in magnetic field by microPET/CT. Picture (A) and sketch (B) of the sample setup in the animal bed of the microPET/CT, and an exemplary microPET/CT image of samples of [68Ga]MNPSNP@THP-1kmPEG in healthy mice (C). Table S1. Estimated average whole organ uptakes (%ID) of lung, liver, spleen and whole bladder (excreted) during the 60 min dynamic PET acquisition. Table S2. Estimated whole organ uptake (%ID) levels of lung, liver, spleen and whole bladder (excreted) at 60 min and 120 min p.i. [file 12951_2023_2041_MOESM1_ESM.docx]

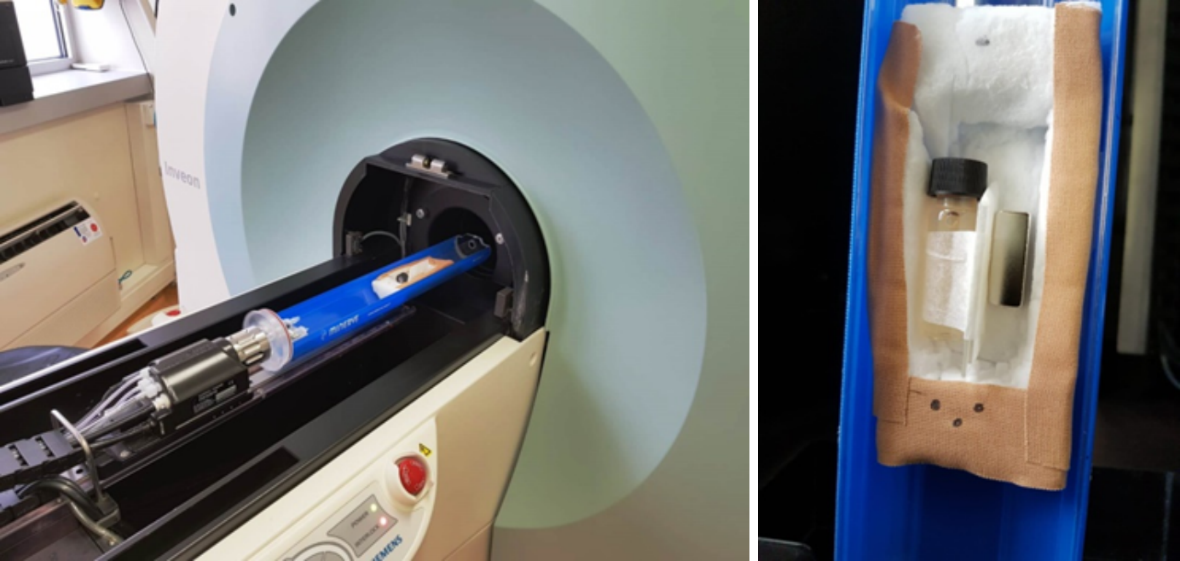


**Figure S1.** Experimental phantom setup for testing the radiolabeled NPs in magnetic field by microPET/CT


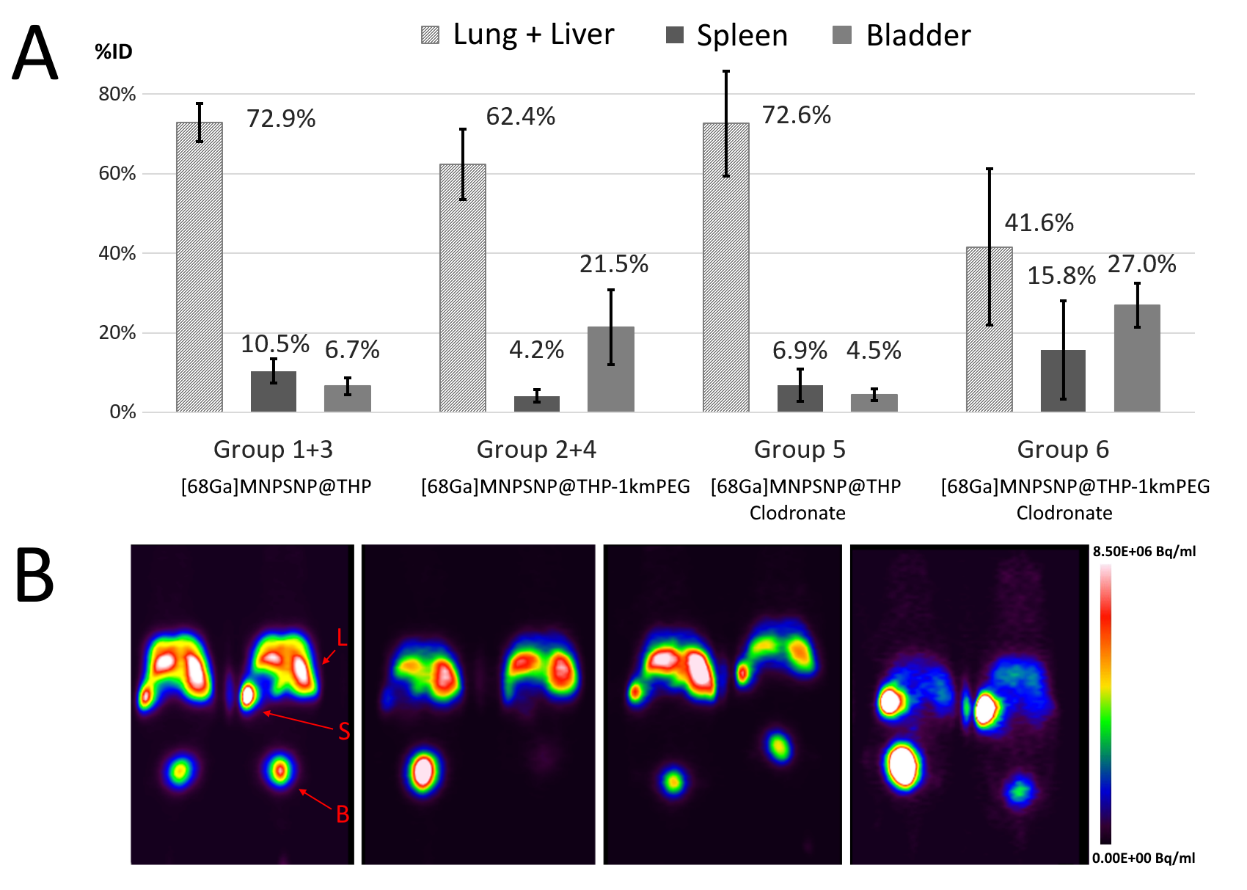


**Figure S2.** Estimated whole organ uptake (%ID) levels of lung, liver, spleen and whole bladder (excreted) (A) and representative PET images (B) of two mice from each group, 60 min post-injection of ^68^Ga-labeled MNPSNP; L = liver, S = spleen, B = bladder.

**
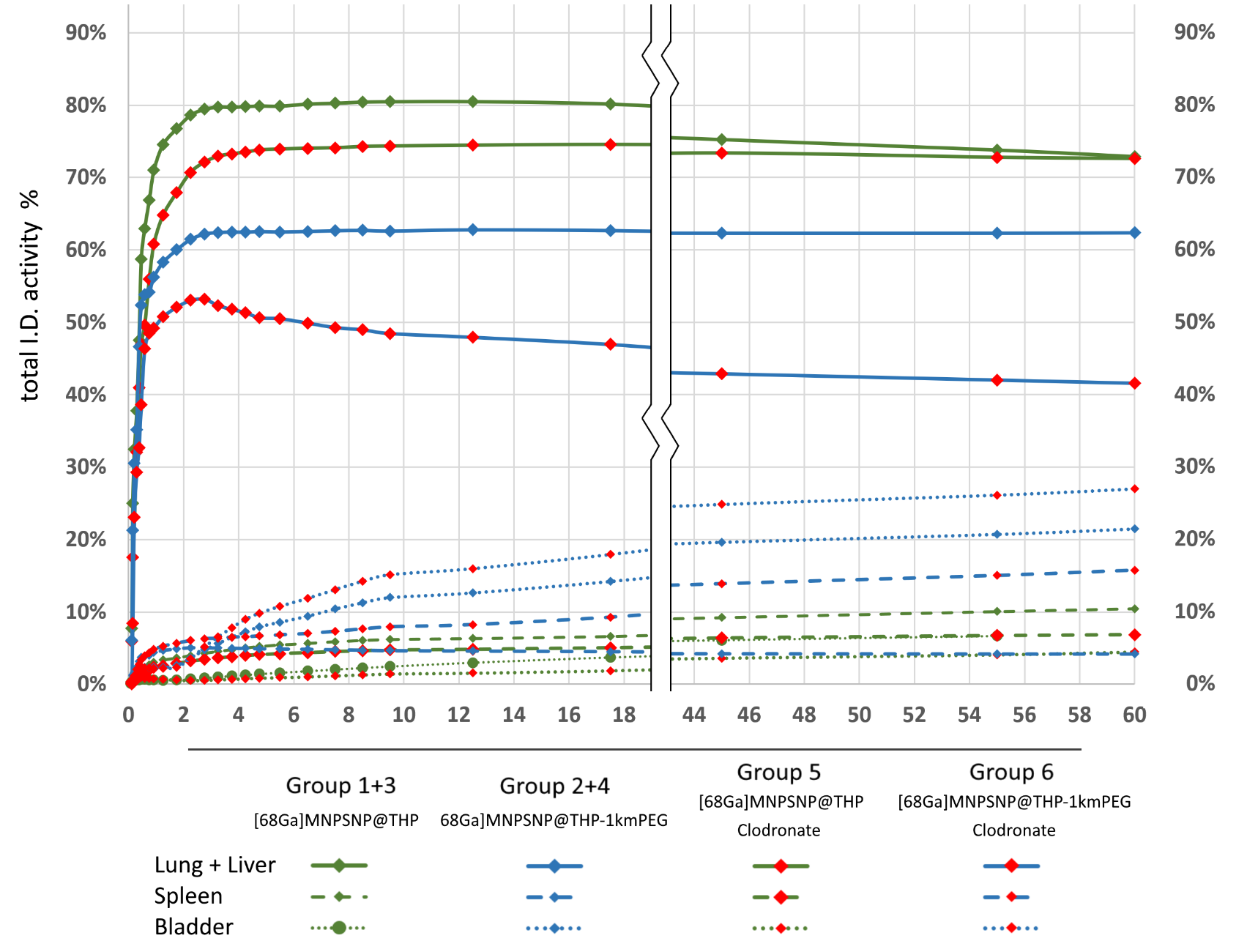
**

**Figure S3.** Estimated whole organ uptake (%ID) levels of lung, liver, spleen and whole bladder (excreted) during the 60 min dynamic PET acquisition


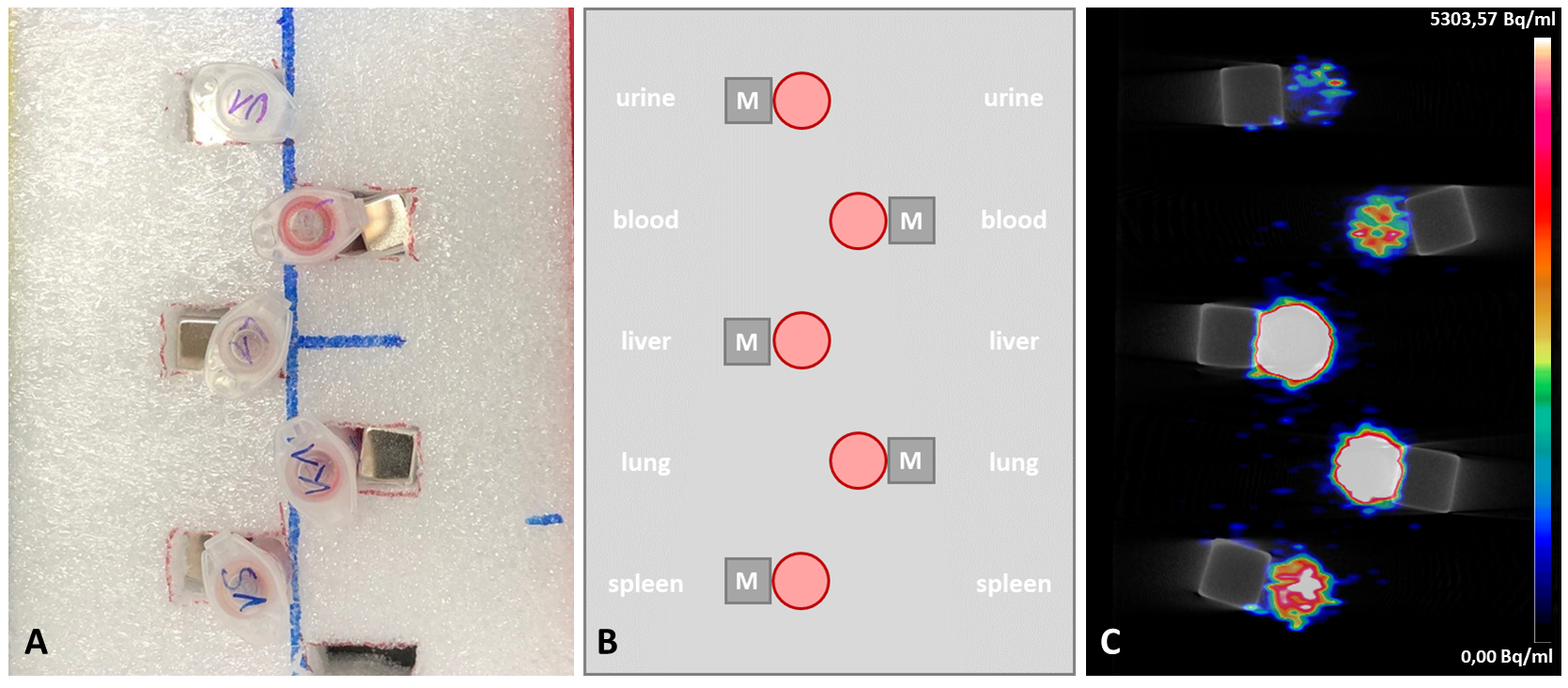


**Figure S4.** Experimental setup and PET/CT images of organ samples and body fluids for detection of *in vivo* metabolites in magnetic field by microPET/CT. Picture (A) and sketch (B) of the sample setup in the animal bed of the microPET/CT, and an exemplary microPET/CT image of samples of [^68^Ga]MNPSNP@THP-1kmPEG in healthy mice (C).

**Table S1.** Estimated average whole organ uptakes (%ID) of lung, liver, spleen and whole bladder (excreted) during the 60 min dynamic PET acquisition

| **Group 1 + 3**  **[^68^Ga]MNPSNP@THP** | | | | | | | | |  | | | **Group 2 + 4**  **[^68^Ga]MNPSNP@THP-1kmPEG** | | | | | | | | |
| --- | --- | --- | --- | --- | --- | --- | --- | --- | --- | --- | --- | --- | --- | --- | --- | --- | --- | --- | --- | --- |
| **Lung + Liver** | | **Spleen** | | | | **Bladder** | | |  | | | **Lung + Liver** | | **Spleen** | | | | **Bladder** | |  |
| **Time (min)** | **AVG** | | **±SD** | **AVG** | **±SD** | | **AVG** | **±SD** | |  | **AVG** | | **±SD** | | **AVG** | **±SD** | **AVG** | | **±SD** |  |
| 0.05 | 0.00% | | 0.00% | 0.00% | 0.00% | | 0.00% | 0.00% | |  | 0.00% | | 0.00% | | 0.00% | 0.00% | 0.00% | | 0.00% |  |
| 0.08 | 0.00% | | 0.00% | 0.00% | 0.00% | | 0.00% | 0.00% | |  | 0.00% | | 0.00% | | 0.00% | 0.00% | 0.00% | | 0.00% |  |
| 0.12 | 7.73% | | 10.01% | 0.14% | 0.16% | | 0.12% | 0.13% | |  | 6.09% | | 9.02% | | 0.22% | 0.38% | 0.53% | | 1.09% |  |
| 0.15 | 25.00% | | 12.81% | 0.51% | 0.48% | | 0.32% | 0.23% | |  | 21.31% | | 12.99% | | 1.15% | 0.91% | 1.30% | | 1.34% |  |
| 0.21 | 32.50% | | 9.51% | 0.83% | 0.45% | | 0.36% | 0.26% | |  | 30.56% | | 7.56% | | 1.62% | 0.95% | 1.23% | | 1.00% |  |
| 0.29 | 37.79% | | 8.29% | 1.21% | 0.36% | | 0.51% | 0.22% | |  | 35.18% | | 6.80% | | 2.33% | 1.10% | 2.05% | | 1.14% |  |
| 0.38 | 47.53% | | 8.83% | 1.61% | 0.49% | | 0.62% | 0.22% | |  | 46.67% | | 8.29% | | 3.14% | 1.41% | 2.49% | | 1.15% |  |
| 0.46 | 58.72% | | 6.44% | 2.07% | 0.56% | | 0.64% | 0.18% | |  | 52.39% | | 6.86% | | 3.69% | 1.48% | 2.33% | | 0.85% |  |
| 0.58 | 62.98% | | 3.93% | 2.37% | 0.61% | | 0.66% | 0.14% | |  | 53.81% | | 5.80% | | 3.88% | 1.39% | 2.36% | | 0.84% |  |
| 0.75 | 66.91% | | 4.86% | 2.71% | 0.75% | | 0.65% | 0.12% | |  | 54.19% | | 5.46% | | 3.99% | 1.39% | 2.29% | | 0.80% |  |
| 0.92 | 71.01% | | 4.74% | 3.06% | 0.90% | | 0.63% | 0.11% | |  | 56.26% | | 5.43% | | 4.38% | 1.50% | 2.33% | | 0.84% |  |
| 1.25 | 74.57% | | 3.72% | 3.34% | 0.98% | | 0.59% | 0.11% | |  | 58.29% | | 5.21% | | 4.67% | 1.65% | 2.31% | | 0.83% |  |
| 1.75 | 76.79% | | 3.32% | 3.64% | 1.11% | | 0.55% | 0.12% | |  | 60.04% | | 5.44% | | 4.90% | 1.70% | 2.61% | | 0.70% |  |
| 2.25 | 78.62% | | 3.52% | 3.96% | 1.22% | | 0.60% | 0.18% | |  | 61.47% | | 6.18% | | 5.06% | 1.63% | 3.65% | | 1.30% |  |
| 2.75 | 79.45% | | 3.72% | 4.29% | 1.33% | | 0.72% | 0.27% | |  | 62.19% | | 6.61% | | 5.05% | 1.58% | 4.73% | | 2.03% |  |
| 3.25 | 79.75% | | 3.83% | 4.55% | 1.43% | | 0.86% | 0.34% | |  | 62.42% | | 7.04% | | 5.01% | 1.59% | 5.66% | | 2.57% |  |
| 3.75 | 79.73% | | 3.83% | 4.79% | 1.52% | | 1.01% | 0.42% | |  | 62.49% | | 7.09% | | 4.98% | 1.60% | 6.51% | | 3.03% |  |
| 4.25 | 79.82% | | 3.84% | 5.02% | 1.62% | | 1.15% | 0.49% | |  | 62.47% | | 7.24% | | 4.94% | 1.60% | 7.27% | | 3.39% |  |
| 4.75 | 79.91% | | 3.92% | 5.23% | 1.71% | | 1.29% | 0.56% | |  | 62.54% | | 7.20% | | 4.92% | 1.64% | 7.95% | | 3.78% |  |
| 5.5 | 79.89% | | 3.90% | 5.44% | 1.77% | | 1.42% | 0.61% | |  | 62.49% | | 7.15% | | 4.88% | 1.61% | 8.59% | | 4.15% |  |
| 6.5 | 80.17% | | 3.95% | 5.66% | 1.88% | | 1.60% | 0.70% | |  | 62.57% | | 7.29% | | 4.85% | 1.62% | 9.42% | | 4.58% |  |
| 7.5 | 80.29% | | 3.96% | 5.89% | 2.00% | | 1.83% | 0.78% | |  | 62.67% | | 7.37% | | 4.77% | 1.59% | 10.41% | | 5.07% |  |
| 8.5 | 80.44% | | 3.97% | 6.07% | 2.06% | | 2.05% | 0.86% | |  | 62.71% | | 7.51% | | 4.71% | 1.60% | 11.28% | | 5.50% |  |
| 9.5 | 80.50% | | 3.99% | 6.20% | 2.11% | | 2.26% | 0.94% | |  | 62.63% | | 7.66% | | 4.66% | 1.57% | 11.98% | | 5.79% |  |
| 12.5 | 80.51% | | 3.95% | 6.33% | 2.15% | | 2.46% | 1.01% | |  | 62.78% | | 7.82% | | 4.60% | 1.57% | 12.63% | | 6.12% |  |
| 17.5 | 80.18% | | 3.76% | 6.62% | 2.12% | | 2.99% | 1.21% | |  | 62.69% | | 7.95% | | 4.51% | 1.59% | 14.21% | | 6.72% |  |
| 22.5 | 79.13% | | 3.64% | 7.12% | 2.00% | | 3.72% | 1.43% | |  | 62.40% | | 8.10% | | 4.41% | 1.60% | 16.08% | | 7.45% |  |
| 27.5 | 77.90% | | 3.66% | 7.70% | 1.93% | | 4.29% | 1.58% | |  | 62.40% | | 8.23% | | 4.32% | 1.62% | 17.44% | | 7.97% |  |
| 35 | 76.76% | | 3.83% | 8.34% | 1.97% | | 4.77% | 1.68% | |  | 62.38% | | 8.38% | | 4.26% | 1.62% | 18.47% | | 8.31% |  |
| 45 | 75.27% | | 4.24% | 9.22% | 2.27% | | 5.38% | 1.81% | |  | 62.32% | | 8.54% | | 4.23% | 1.62% | 19.62% | | 8.78% |  |
| 55 | 73.83% | | 4.66% | 10.06% | 2.80% | | 6.10% | 1.95% | |  | 62.32% | | 8.72% | | 4.20% | 1.61% | 20.72% | | 9.24% |  |
| 60 | 72.92% | | 4.77% | 10.45% | 3.00% | | 6.67% | 2.09% | |  | 62.38% | | 8.79% | | 4.20% | 1.59% | 21.47% | | 9.47% |  |

| **Group 5**  **[^68^Ga]MNPSNP@THP + CLODRONATE** | | | | | | | | |  | | | **Group 6**  **[^68^Ga]MNPSNP@THP-1kmPEG + CLODRONATE** | | | | | | | | |
| --- | --- | --- | --- | --- | --- | --- | --- | --- | --- | --- | --- | --- | --- | --- | --- | --- | --- | --- | --- | --- |
| **Lung + Liver** | | **Spleen** | | | | **Bladder** | | |  | | | **Lung + Liver** | | **Spleen** | | | | **Bladder** | |  |
| **Time (min)** | **AVG** | | **±SD** | **AVG** | **±SD** | | **AVG** | **±SD** | |  | **AVG** | | **±SD** | | **AVG** | **±SD** | **AVG** | | **±SD** |  |
| 0.05 | 0.00% | | 0.00% | 0.00% | 0.00% | | 0.00% | 0.00% | |  | 0.00% | | 0.00% | | 0.00% | 0.00% | 0.00% | | 0.00% |  |
| 0.08 | 0.00% | | 0.00% | 0.00% | 0.00% | | 0.00% | 0.00% | |  | 0.00% | | 0.00% | | 0.00% | 0.00% | 0.00% | | 0.00% |  |
| 0.12 | 5.90% | | 8.34% | 0.09% | 0.13% | | 0.46% | 0.53% | |  | 0.34% | | 0.59% | | 0.00% | 0.00% | 0.01% | | 0.03% |  |
| 0.15 | 17.57% | | 12.89% | 0.23% | 0.30% | | 0.59% | 0.24% | |  | 8.43% | | 8.60% | | 0.40% | 0.57% | 0.24% | | 0.40% |  |
| 0.21 | 23.09% | | 9.41% | 0.39% | 0.43% | | 0.83% | 0.44% | |  | 23.08% | | 7.16% | | 1.14% | 1.08% | 0.86% | | 0.71% |  |
| 0.29 | 32.06% | | 13.65% | 0.74% | 0.49% | | 0.90% | 0.52% | |  | 29.30% | | 6.11% | | 2.08% | 1.15% | 1.54% | | 1.11% |  |
| 0.38 | 41.00% | | 11.89% | 1.13% | 0.69% | | 0.80% | 0.38% | |  | 32.69% | | 9.31% | | 2.74% | 1.60% | 1.78% | | 1.37% |  |
| 0.46 | 47.02% | | 10.25% | 1.38% | 0.76% | | 0.95% | 0.46% | |  | 38.64% | | 10.06% | | 3.38% | 1.75% | 2.00% | | 1.35% |  |
| 0.58 | 49.62% | | 10.19% | 1.59% | 0.79% | | 0.80% | 0.34% | |  | 46.36% | | 12.25% | | 3.90% | 1.72% | 2.28% | | 1.26% |  |
| 0.75 | 55.98% | | 10.04% | 1.99% | 0.95% | | 0.80% | 0.34% | |  | 48.51% | | 6.82% | | 4.38% | 1.53% | 2.25% | | 1.09% |  |
| 0.92 | 60.79% | | 9.65% | 2.31% | 1.09% | | 0.76% | 0.31% | |  | 49.21% | | 6.63% | | 4.83% | 1.58% | 2.20% | | 1.15% |  |
| 1.25 | 64.79% | | 10.88% | 2.57% | 1.21% | | 0.69% | 0.30% | |  | 50.78% | | 7.58% | | 5.26% | 1.63% | 2.18% | | 1.15% |  |
| 1.75 | 67.95% | | 11.25% | 2.88% | 1.38% | | 0.58% | 0.24% | |  | 52.12% | | 8.86% | | 5.68% | 1.76% | 2.34% | | 1.04% |  |
| 2.25 | 70.72% | | 11.64% | 3.21% | 1.60% | | 0.52% | 0.17% | |  | 53.04% | | 9.81% | | 6.04% | 1.93% | 3.55% | | 1.06% |  |
| 2.75 | 72.10% | | 11.88% | 3.43% | 1.79% | | 0.55% | 0.13% | |  | 53.22% | | 10.49% | | 6.31% | 2.13% | 5.15% | | 1.27% |  |
| 3.25 | 72.94% | | 12.09% | 3.65% | 1.94% | | 0.62% | 0.15% | |  | 52.33% | | 11.33% | | 6.35% | 2.20% | 6.55% | | 1.40% |  |
| 3.75 | 73.26% | | 12.29% | 3.82% | 2.07% | | 0.69% | 0.19% | |  | 51.80% | | 11.61% | | 6.51% | 2.38% | 7.82% | | 1.57% |  |
| 4.25 | 73.50% | | 12.32% | 3.97% | 2.18% | | 0.77% | 0.24% | |  | 51.36% | | 12.12% | | 6.57% | 2.42% | 8.99% | | 1.82% |  |
| 4.75 | 73.80% | | 12.37% | 4.10% | 2.31% | | 0.85% | 0.30% | |  | 50.65% | | 12.47% | | 6.66% | 2.52% | 9.82% | | 1.96% |  |
| 5.5 | 73.94% | | 12.42% | 4.23% | 2.42% | | 0.93% | 0.35% | |  | 50.51% | | 12.85% | | 6.82% | 2.67% | 10.77% | | 2.14% |  |
| 6.5 | 74.03% | | 12.51% | 4.37% | 2.57% | | 1.03% | 0.42% | |  | 49.92% | | 13.10% | | 7.02% | 2.85% | 11.87% | | 2.41% |  |
| 7.5 | 74.10% | | 12.51% | 4.52% | 2.76% | | 1.17% | 0.50% | |  | 49.29% | | 13.58% | | 7.32% | 3.23% | 13.08% | | 2.68% |  |
| 8.5 | 74.28% | | 12.50% | 4.64% | 2.93% | | 1.30% | 0.56% | |  | 48.99% | | 14.02% | | 7.64% | 3.54% | 14.24% | | 2.92% |  |
| 9.5 | 74.35% | | 12.45% | 4.74% | 3.07% | | 1.43% | 0.64% | |  | 48.46% | | 14.34% | | 7.93% | 3.91% | 15.15% | | 3.12% |  |
| 12.5 | 74.45% | | 12.38% | 4.83% | 3.19% | | 1.55% | 0.70% | |  | 47.94% | | 14.64% | | 8.24% | 4.27% | 15.98% | | 3.26% |  |
| 17.5 | 74.56% | | 12.26% | 5.05% | 3.48% | | 1.87% | 0.83% | |  | 46.96% | | 15.35% | | 9.27% | 5.32% | 17.93% | | 3.62% |  |
| 22.5 | 74.41% | | 12.44% | 5.31% | 3.74% | | 2.36% | 1.00% | |  | 45.54% | | 16.30% | | 10.71% | 6.85% | 20.20% | | 3.96% |  |
| 27.5 | 73.89% | | 12.68% | 5.65% | 3.81% | | 2.78% | 1.12% | |  | 44.51% | | 17.07% | | 11.86% | 8.12% | 21.93% | | 4.37% |  |
| 35 | 72.99% | | 12.50% | 5.97% | 3.88% | | 3.12% | 1.17% | |  | 43.74% | | 17.63% | | 12.74% | 9.09% | 23.30% | | 4.80% |  |
| 45 | 73.37% | | 13.60% | 6.40% | 3.88% | | 3.60% | 1.31% | |  | 42.90% | | 18.43% | | 13.89% | 10.34% | 24.85% | | 5.16% |  |
| 55 | 72.78% | | 13.33% | 6.73% | 4.00% | | 4.07% | 1.42% | |  | 42.04% | | 19.15% | | 15.04% | 11.59% | 26.14% | | 5.46% |  |
| 60 | 72.62% | | 13.14% | 6.86% | 4.03% | | 4.46% | 1.47% | |  | 41.59% | | 19.59% | | 15.77% | 12.38% | 27.00% | | 5.55% |  |

**Table S2.** Estimated whole organ uptake (%ID) levels of lung, liver, spleen and whole bladder (excreted) at 60 min and 120 min p.i.

| **Group 1 + 3 - [^68^Ga]MNPSNP@THP** | | | | | | |
| --- | --- | --- | --- | --- | --- | --- |
|  | Lung + Liver | | Spleen |  | Bladder |  |
| Time int. | AVG | ±SD | AVG | ±SD | AVG | ±SD |
| 60 min | 72.92% | 4.77% | 10.45% | 3.00% | 6.67% | 2.09% |
| 120 min | 70.27% | 4.87% | 10.77% | 3.00% | 9.80% | 2.72% |
|  |  |  |  |  |  |  |
| **Group 2 + 4 - [^68^Ga]MNPSNP@THP-1kmPEG** | | | | | | |
|  | Lung + Liver | | Spleen |  | Bladder |  |
| Time int. | AVG | ±SD | AVG | ±SD | AVG | ±SD |
| 60 min | 62.38% | 8.79% | 4.20% | 1.59% | 21.47% | 9.47% |
| 120 min | 63.07% | 5.02% | 6.33% | 2.73% | 4.65% | 0.77% |
|  |  |  |  |  |  |  |
| **Group 5 - [^68^Ga]MNPSNP@THP + CLODRONATE** | | | | | | |
|  | Lung + Liver | | Spleen |  | Bladder |  |
| Time int. | AVG | ±SD | AVG | ±SD | AVG | ±SD |
| 60 min | 72.62% | 13.14% | 6.86% | 4.03% | 4.46% | 1.47% |
| 120 min | 61.07% | 10.31% | 4.35% | 1.51% | 6.91% | 6.52% |
|  |  |  |  |  |  |  |
| **Group 6 - [^68^Ga]MNPSNP@THP-1kmPEG + CLODRONATE** | | | | | | |
|  | Lung + Liver | | Spleen |  | Bladder |  |
| Time int. | AVG | ±SD | AVG | ±SD | AVG | ±SD |
| 60 min | 41.59% | 19.59% | 15.77% | 12.38% | 27.00% | 5.55% |
| 120 min | 37.26% | 22.28% | 11.19% | 10.50% | 22.37% | 1.67% |
